# Topological flat bands in frustrated kagome lattice CoSn

**DOI:** 10.1038/s41467-020-17465-1

**Published:** 2020-08-10

**Authors:** Mingu Kang, Shiang Fang, Linda Ye, Hoi Chun Po, Jonathan Denlinger, Chris Jozwiak, Aaron Bostwick, Eli Rotenberg, Efthimios Kaxiras, Joseph G. Checkelsky, Riccardo Comin

**Affiliations:** 1 Department of Physics, Massachusetts Institute of Technology, Cambridge, MA 02139 USA; 2 Department of Physics, Harvard University, Cambridge, MA 02138 USA; 3 John A. Paulson School of Engineering and Applied Science, Harvard University, Cambridge, MA 02138 USA; 4 Department of Physics and Astronomy, Center for Materials Theory, Rutgers University, Piscataway, NJ 08854 USA; 5 Advanced Light Source, E. O. Lawrence Berkeley National Laboratory, Berkeley, CA 94720 USA

**Keywords:** Electronic properties and materials, Topological insulators

## Abstract

Electronic flat bands in momentum space, arising from strong localization of electrons in real space, are an ideal stage to realize strongly-correlated phenomena. Theoretically, the flat bands can naturally arise in certain geometrically frustrated lattices, often with nontrivial topology if combined with spin-orbit coupling. Here, we report the observation of topological flat bands in frustrated kagome metal CoSn, using angle-resolved photoemission spectroscopy and band structure calculations. Throughout the entire Brillouin zone, the bandwidth of the flat band is suppressed by an order of magnitude compared to the Dirac bands originating from the same orbitals. The frustration-driven nature of the flat band is directly confirmed by the chiral *d*-orbital texture of the corresponding real-space Wannier functions. Spin-orbit coupling opens a large gap of 80 meV at the quadratic touching point between the Dirac and flat bands, endowing a nonzero Z_2_ invariant to the flat band. These findings demonstrate that kagome-derived flat bands are a promising platform for novel emergent phases of matter at the confluence of strong correlation and topology.

## Abstract (web summary)

The experimental realization of lattice-born flat bands with nontrivial topology has been elusive. Here, the authors observe topological flat bands near the Fermi level in a kagome metal CoSn, with flat bands as well as Dirac bands originating from 3d orbitals in a frustrated kagome geometry.

## Introduction

Electronic correlations are a hallmark of condensed matter systems with many-body character. Localizing electrons in real space is often considered as a route to enhance correlation effects and engineer emergent phases of matter. Most well-known examples include *d*-electron systems, where the subtle balance between kinetic energy and localization-enhanced Coulomb interaction leads to collective electron behavior and rich many-body physics encompassing unconventional superconductivity, metal–insulator transitions, density-wave instabilities, and quantum spin liquids^1^. In band-like systems, electrons can still be confined in real space in lattices supporting dispersionless electronic excitations (i.e., flat bands) in momentum space. Due to the prominence of the interaction energy scale over the quenched kinetic energy, flat bands represent a versatile platform to explore exotic correlated electron phenomena. Notable examples include *f*-electron systems with Kondo physics and heavy fermions^2^, Landau levels under high magnetic fields and the fractional quantum Hall effect^3^, and, more recently, magic-angle twisted bilayer graphene superlattices with Mott-insulating phase and unconventional superconductivity^4,5^.

**Fig. 1:**
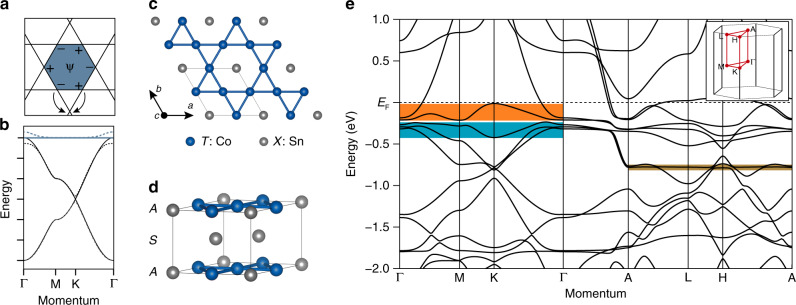
Electronic confinement and flat band in ideal kagome lattice and kagome metal CoSn. **a** Confinement of electron in kagome lattice with nearest-neighbor hopping. Plus and minus signs indicate the phase of flat band eigenstate at neighboring sublattices. Any hoppings outside the hexagon (arrows) are canceled by destructive quantum interferences, resulting in the perfect localization of electron in the blue-colored hexagon. **b** Tight-binding band structure of kagome lattice featuring flat band (blue solid line) and two Dirac bands with linear crossing at K (black solid lines). Inclusion of spin–orbit coupling gaps both Dirac crossing and quadratic touching between the flat band and the Dirac band (dotted lines). **c** In-plane structure of kagome layer in CoSn consists of kagome network of Co atoms and space-filling Sn atoms. **d** Three-dimensional structure of CoSn with alternating stacking of the kagome layer *A* and Sn layer *S*. **e** Relativistic density functional theory (DFT) band structure of CoSn. Orange, cyan, and brown-colored regions highlight the manifestation of the kagome flat band flat bands with different *d*-orbital characters. Inset shows the bulk Brillouin zone of CoSn. The DFT Fermi level is shifted down by 140 meV to fit the experimental Fermi level.

A known experimental route to engineering electronic confinement and flat bands relies on the destructive quantum phase interference of fermion hopping paths in certain networks, including the dice, Lieb, kagome, and decorated square lattices^6–12^. Here we focus on the case of the kagome lattice depicted in Fig. 1a. In the simplest nearest-neighbor electronic hopping model of the *s*-orbital kagome lattice *H* = Σ_<*i,j*>_*c*${\,\!}_{i}^{\dagger}$*c*_*j*_ + h.c., one can construct real-space eigenfunctions with alternating phases at neighboring corners of the hexagon (Fig. 1a). This electronic state is geometrically confined within the single hexagon since any hopping to neighboring cells is hindered by the destructive phase interference as shown in Fig. 1a. This real-space electronic localization translates into momentum–space (Bloch) eigenfunctions with no energy dispersion, namely flat bands (Fig. 1b). In the tight-binding model of kagome lattice, this dispersionless excitation materializes alongside a pair of Dirac bands that are protected by the lattice symmetry similar to the case of the honeycomb lattice. Both the linear band crossing at K and quadratic band touching point at Γ become gapped once spin–orbit coupling (SOC) is included, and the Dirac and flat bands become topologically nontrivial^10–15^. This peculiar band structure of the kagome lattice has recently attracted significant interest, not only in the context of electronic topology—topological insulator, Chern insulator, and fractional quantum Hall phases^10–15^—but also as a platform to realize many-body electronic phases—density waves, magnetism, Pomeranchuk instability, and superconductivity^16–18^.

While the lattice-born flat bands have been recently observed in optical and engineered atomic Lieb lattices, their experimental realization in a solid-state system has remained elusive^19,20^. Unlike the ideal case shown in Fig. 1b, the dispersion of the flat band in real kagome compounds is modified by additional factors, such as in-plane next-nearest-neighbor hopping, interlayer coupling, and multiple orbital degrees of freedom. Therefore, the experimental realization of the kagome flat band requires careful and systematic material design. Prior scanning tunneling microscopy (STM) studies on kagome compounds Fe_3_Sn_2_ and Co_3_Sn_2_S_2_ reported the evidence of flat bands from the enhancement of the momentum-integrated density of states (DOS)^21–23^. However, detailed band structure calculations revealed that the region of existence of these dispersionless excitations is rather limited in the momentum space^21,24^, due to complex hopping pathways distorting the flat dispersions in these compounds. These calculations are consistent with the relatively weak enhancement of the DOS in STM measurements compared to the ideal flat band case (see the Supplementary Fig. 1 for details)^21–23^. Accordingly, angle-resolved photoemission spectroscopy (ARPES) experiments have been carried out on Fe_3_Sn_2_ and Co_3_Sn_2_S_2_, but no robust evidence for flat bands have been detected^24,25^. The unambiguous momentum–space identification of the kagome-based flat band and analysis of its topological character have, therefore, remained the subject of ongoing investigations.

In the present study of kagome metal CoSn, we combine ARPES and band structure calculations to report the presence of topological flat bands with suppressed bandwidth in all three momentum–space directions. CoSn belongs to the family of binary kagome metals *T*_*m*_*X*_*n*_ (*T*: 3*d* transition metals, *X*: Sn, Ge, *m*:*n* = 3:1, 3:2, 1:1), wherein the kagome network is constructed upon 3*d* transition metals as shown in Fig. 1c. Compounded with the variety of magnetic ground states and topological electronic structures, this material family has been recently spotlighted as a versatile platform for novel correlated topological phases^25–29^. For example, previous studies on Mn_3_(Sn/Ge), Fe_3_Sn_2_, and FeSn have revealed band singularities ranging from three-dimensional Weyl points to two-dimensional (2D) Dirac points, which, in combination with intrinsic magnetism, generate large and intrinsic anomalous Hall conductivity^25–29^. Among the series, *TX* compounds (*m*:*n* = 1:1) have been suggested as viable hosts for the prototypical kagome electronic structure because of their spatially decoupled quasi-2D kagome planes (Fig. 1d)^26^. In FeSn, for example, the kagome-derived 2D Dirac band structure has been observed by ARPES, as well as a dispersionless band below the Fermi level, whose relationship with the observed Dirac bands has not be resolved^26^. Consequently, the presence of a gap at the quadratic band touching points between the Dirac and flat bands could not be examined, despite its central role for the topological character of the flat band. The replacement of Co at the transition metal site suppresses the formation of local moments and magnetic ordering in this compound, presumably due to a higher *d*-orbital filling^30^, while, at the same time, it shifts the overall band structure below the Fermi energy, so that all kagome-derived electronic excitations (both Dirac the flat bands) can be accessed by ARPES. Consequently, we could directly visualize the kagome-derived flat band as well as the large SOC gap at the quadratic band touching point between Dirac and flat bands, which endows nontrivial topology to the flat band as long predicted theoretically.

## Results

### Visualization of kagome flat bands using ARPES

**Fig. 2:**
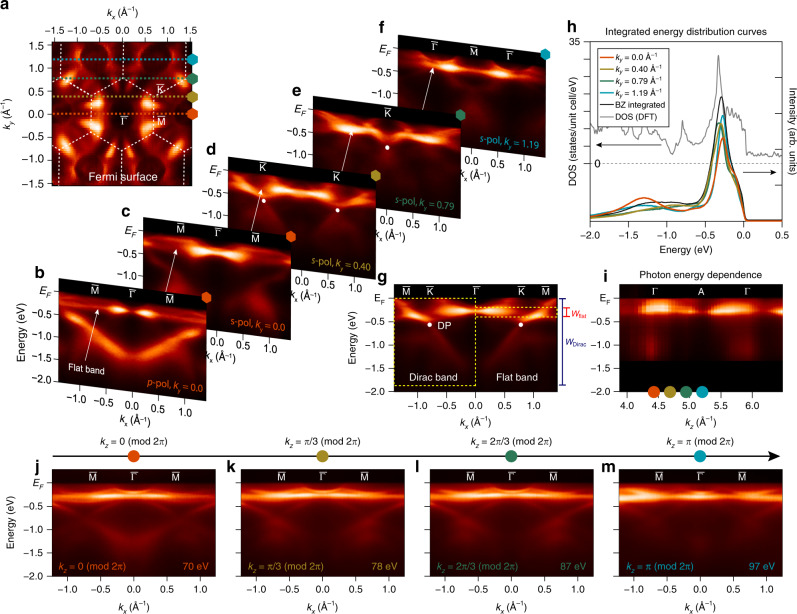
Direct visualization of flat band in CoSn. **a** Fermi surface of CoSn measured with 92 eV photons. Hexagonal surface Brillouin zones are marked with white dashed lines. **b**–**f** Energy–momentum dispersion of CoSn measured at *k*_*y*_ = 0.0 Å^−1^ (**b**, **c**), 0.40 Å^−1^ (**d**), 0.79 Å^−1^ (**e**), and 1.19 Å^−1^ (**f**). **g** Energy–momentum dispersion of CoSn measured along ${\bar{\Gamma}}$–${\bar{\mathrm{K}}}$–${\bar{\mathrm{M}}}$ high-symmetry direction. The Dirac points at ${\bar{\mathrm{K}}}$ are marked with white dots in (**c**, **d**, **g**). Red and blue scales indicate the bandwidth of the Dirac and flat bands, respectively. **h** Momentum-integrated energy distribution curves from spectra in **c**–**f** (orange, brown, green, and cyan lines, respectively), and from the entire first Brillouin zone (black line). Density of states curve from the density functional theory calculation of CoSn (gray line) is also overlaid. **i** Out-of-plane dispersion of the flat band from photon-energy-dependent ARPES experiment with the photon energy tuned from 50 to 155 eV. **j**–**m** Energy–momentum dispersion of the flat band along ${\bar{\Gamma}}$–${\bar{\mathrm{M}}}$ high-symmetry direction measured at representative *k*_*z*_ = 0, *π*/3, 2*π*/3, and *π* (mod 2*π*), respectively.

Figure 2 summarizes the experimental band structure of CoSn as measured using ARPES. The data in Fig. 2a–h were acquired using 92 eV photoexcitation, which maximizes the signal from kagome band structures. Figure 2a shows the Fermi surface of CoSn and its characteristic hexagonal symmetry (the surface Brillouin zone is marked by the white dashed lines) as expected from the underlying symmetry of the kagome lattice. In Fig. 2b–f we display a series of energy–momentum dispersions measured at *k*_*y*_ = 0.0, 0.40, 0.79, and 1.19 Å^−1^ (corresponding to orange, brown, green, and cyan traces in Fig. 2a, respectively) across various high-symmetry points. As shown in Fig. 2b, c, the energy–momentum dispersion along the ${\bar{Γ}}$–${\bar{\mathrm{M}}}$ high-symmetry direction displays a strikingly nondispersive band near the Fermi level at −0.27 ± 0.05 eV, which manifests itself independently of photon polarization. The dispersion of the flat band in this specific direction is lower than the experimental broadening of the band, which is below 50 meV. As shown in Fig. 2d–f, the nondispersive nature of the flat band spans most of the Brillouin zone, and acquires a small dispersion only close to the ${\bar{\mathrm{K}}}$ point. We note that the acquisition of a small but finite bandwidth near the ${\bar{\mathrm{K}}}$ point is typical for realistic kagome models due to next-nearest-neighbor hopping^14,22,31^. Even using conservative estimates, the bandwidth of the flat band over the entire Brillouin zone does not exceed 150 meV, suggesting that the electron kinetic energy is strongly quenched by quantum interference effects, preventing delocalization of the wave function across the lattice.

To further examine the lack of dispersion of the flat band, we present in Fig. 2g the experimental band structure measured along the ${\bar{\Gamma}}$–${\bar{\mathrm{K}}}$–${\bar{\mathrm{M}}}$ high-symmetry direction. Near the K point, a linearly dispersing Dirac band is found (see also Fig. 2d, e and Supplementary Fig. 2 for further characterization of the Dirac bands), which is also characteristic of the kagome band structure as previously observed in Fe_3_Sn_2_ and FeSn^25,26^. The Dirac point is located at −0.57 ± 0.05 eV, and only one branch of the Dirac cone could be observed along ${\bar{\Gamma}}$–${\bar{\mathrm{K}}}$ direction due to the matrix element effect associated to the chirality of the Dirac fermion similar to graphene^26,32^. The velocity of the Dirac band is (1.8 ± 0.1) × 10^5^ m/s, which is renormalized by 23% from the density functional theory (DFT) value (see below) similar to magnetic 3*d* metals Fe and Ni^33,34^. With the yellow dashed boxes and red and blue bars of Fig. 2g, we directly compare the bandwidth of the Dirac and flat bands. The bandwidth of Dirac band extends over a range of 1.5 eV, which is typical for 3*d*-electron systems, such as elemental transition metals and cuprates^35–37^. In contrast, the reduced bandwidth of the flat band (<0.15 eV) is highly unusual, and can be regarded as a direct consequence of quantum phase interference effects in the kagome lattice as introduced in Fig. 1a.

A defining trait of the flat band is a diverging DOS, which often sets the stage for emergent electronic phases characterized by collective electronic, magnetic, and superconducting orders^16–18^. The high DOS from the flat band in CoSn can be captured (if we neglect the photoemission matrix element effect) from the momentum-integrated energy distribution curves shown in Fig. 2h. Here, colored lines are the momentum-integrated spectra from the energy–momentum sections in Fig. 2c–f, while the black line is obtained by integration over the full first Brillouin zone. All integrated energy distribution curves show a sharp and intense peak near the fixed flat band energy −0.27 ± 0.05 eV), reflecting the high DOS associated to the nondispersive flat band. In contrast, the spectral weight from all other bands (including the Dirac bands) is spread out in energy and further modulated as a function of *k*_*y*_.

In Fig. 2i–m we present the evolution of the flat band as a function of out-of-plane momentum *k*_*z*_ as measured using photon-energy-dependent ARPES. The flat band displays negligible dispersion (<50 meV) along Γ–A as shown in Fig. 2i, while fully retaining its in-plane flatness as shown in the representative cuts of Fig. 2j–m, measured at *k*_*z*_ = 0, *π*/3, 2*π*/3, and *π* (mod 2*π*). The flat band can thus be considered flat also along the *k*_*z*_ direction. Even though the bandwidth-canceling mechanisms are different for the in-plane and out-of-plane directions—the former is quenched by quantum phase interference, while the latter is suppressed by virtue of quasi-2D layered structure (see Fig. 1d)—the flat dispersion is realized along all three momentum directions in CoSn. This is indeed at variance with the limited momentum range of the flat bands in previously studied kagome compounds^21–24^. Accordingly, the kinetic energy of flat band electrons in CoSn is strongly quenched, and interaction-driven many-body ground states are naturally expected once this lattice-born flat band is tuned to the Fermi level. In other words, if we start from a simple Hubbard model *H* = −*t*Σ_<*i,j*>_*c*${\,\!}_{i}^{\dagger}$*c*_*j*_ + *U*Σ_*i*_*n*_*i*↑_*n*_*i*↓_, where *t* is the hopping integral and *U* is the on-site interaction, the large *U*/*t* value essential to promote strong electronic correlations can be attained even with relative small *U* value owing to the quenched *t* of the flat bands. We calculate *U* ≈ 5–6 eV for Co 3*d* electrons, based on the linear response approach^38^ (see Methods), confirming that the interaction energy scale indeed dominates the kinetic energy scale of the flat band electrons as measured by the quenched bandwidth of the flat bands (<0.2 eV). The realization of strongly correlated phases based on the flat band electrons has been recently demonstrated in magic-angle twisted Moiré superlattices, whose flat minibands serve as a basis for Mott-insulating, superconducting, and magnetic ground states^4,5,39^. Similar correlated states of matter have been also theoretically investigated based on the flat band of the kagome lattice^16,17,40–43^. Here, we experimentally identify a candidate material to extend this research avenue to real kagome systems.

### DFT calculations and Wannier function analysis

To understand the origin of the observed flat band, we use relativistic DFT to calculate the theoretical band structure of CoSn as shown in Fig. 1e. Despite the complexity inherent to the presence of multiple *d*-orbital degrees of freedom, the calculations closely capture the experimental manifestation of the kagome band structure. For example, in the *k*_*z*_ = 0 plane, a strongly dispersing Dirac bands (with Dirac points at K) is reproduced between −0.3 and −1.8 eV with a 1.1–1.5 eV bandwidth. At the same time, two flat bands (with bandwidth quenched below 0.2 eV) appear above the Dirac bands (highlighted with orange and cyan boxes), in close correspondence to Fig. 1b. Our analysis of orbital character in Supplementary Fig. 3 reveals that the two flat bands arise from different orbital degrees of freedom: the upper (lower) flat band in the orange (cyan) box is mainly formed by *d*_*xy*_/*d*_*x*2 *−y*2_ (*d*_*xz*_/*d*_*yz*_) orbitals. These flat bands also have suppressed dispersion along *k*_*z*_ direction (see Γ–A direction for example), consistent with experimental observations. We also found a flat band at *k*_*z*_ = *π* (brown box in Fig. 1e), which arises from *d*_*z*2_ orbital. However, due to its out-of-plane orbital character, the band is not effectively localized in the *k*_*z*_ direction, exhibiting a bandwidth >1 eV. We note that the steep *k*_*z*_ dispersive bands may account for the low out-of-plane resistivity and positive out-of-plane Hall coefficient observed in our transport measurements on CoSn (see the Supplementary Note 1 and Supplementary Fig. 4).

**Fig. 3:**
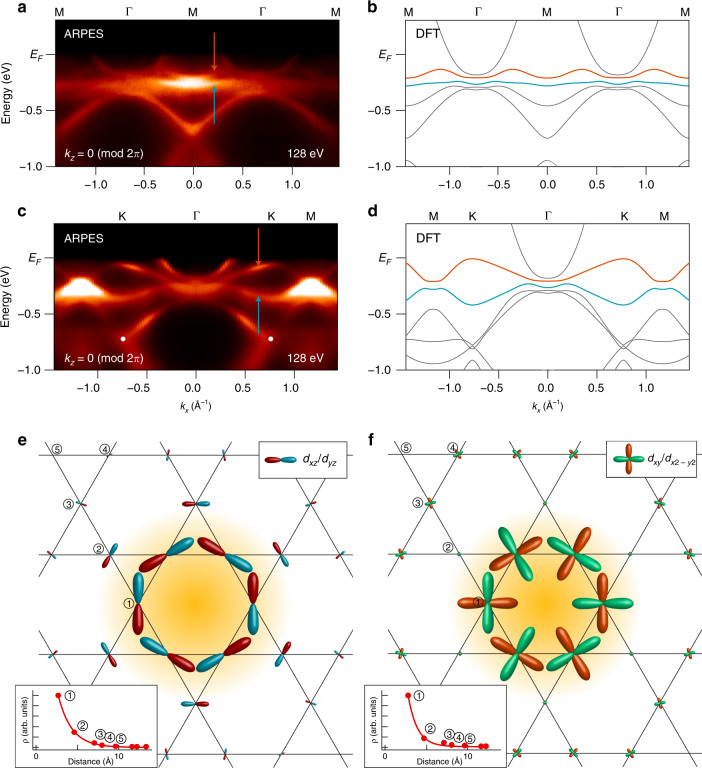
Orbital texture of flat bands and effective Wannier wave function. **a**, **c** High-resolution band structure of CoSn along Γ–M and Γ–K–M high-symmetry directions, respectively. The data are measured with 128 eV photons, which probes band structure at *k*_*z*_ = 0 plane. **b**, **d** Corresponding DFT band structures. Cyan and orange lines, respectively, mark two flat bands arising from *d*_*xz*_/*d*_*yz*_ and *d*_*xy*_/*d*_*x*2 *−y*2_ orbital degrees of freedom. Experimental dispersion of the flat bands (marked with orange and cyan arrows in **a**, **c**) are well reproduced by the calculation. The Dirac point at K are marked with white dots in **c**. **e**, **f** Orbital textures of the effective Wannier states constructed from the flat bands with *d*_*xz*_/*d*_*yz*_ and *d*_*xy*_/*d*_*x*2 *−y*2_ orbitals, respectively. Length scale of the orbitals at each site is proportional to the orbital wave functions in the majority spin channel. Insets of **e**, **f** display the decay of total charge density (including the contribution from other orbitals) of the Wannier functions away from the central hexagon.

For a detailed comparison between experiment and theory, we tune the photon energy to 128 eV to visualize the in-plane electronic structure at the *k*_*z*_ = 0 (mod 2*π*) plane and acquire high-resolution energy–momentum maps of the flat band. As shown in Fig. 3a, c, we could directly detect the two flat bands with dispersion <0.1 eV along Γ–M and <0.2 eV along Γ–K–M. The experimental dispersion closely follows the theoretical dispersion shown in Fig. 3b, d (which are shifted up by 140 meV to match the experimental Fermi level), confirming the assignment of these features to the two flat bands arising from different *d*-orbital degrees of freedom as discussed above. The Dirac point is again observed at K, and positioned at slightly higher binding energy 0.73 ± 0.05 eV due to small but finite dispersion of the Dirac bands along *k*_*z*_. We note that the flat band highlighted in Fig. 2 corresponds to the lower flat band (cyan) with *d*_*xz*_/*d*_*yz*_ orbital characters. Below, we will focus on these prototypical flat bands at *k*_*z*_ = 0 to analyze their localization and topology.

At this point, an important outstanding question is how the localization mechanism in the simple *s*-orbital kagome tight-binding model (Fig. 1a, b) manifests in the realistic *d*-orbital kagome lattice of CoSn. To address this aspect, we derived a DFT-based ab initio tight-binding model of CoSn (Supplementary Fig. 5), and use the *k*_*z*_ = 0 flat bands to construct the real-space effective Wannier functions on the 2D kagome plane (see the Supplementary Note 3 for details). We construct the flat band Wannier function to retain the highest degree of symmetries possible (except those abandoned by the topological obstructions associated with nontrivial *Z*_2_ invariant and mirror Chern number; see “Discussion” below), which include a subset of important symmetries of the kagome lattice such as *C*_*6*_ rotational symmetry, *xz*/*yz* mirror symmetry, combined inversion/time-reversal symmetry, and combined *xy* mirror/time-reversal symmetry. As such, the Wannier functions we derived could serve as a basis for future analyses of interaction effects within the flat bands. The real-space orbital textures of the constructed flat band Wannier functions are displayed in Fig. 3e, f, while the corresponding spin textures are displayed in Supplementary Fig. 6. Several important points are apparent: (1) Due to the finite dispersion of the flat bands in CoSn, we could observe a finite charge density leaking out of the central hexagon unlike the ideal case in Fig. 1a. Nonetheless, the charge density rapidly and exponentially decays away from the central hexagon (insets in Fig. 3e, f), and 85% of total charge are confined in the first to third sites from the center. This provides the length scale of the localization ≈7 Å. (2) If we focus on the states at the central hexagon, chiral orbital textures could be observed around the hexagon, in which the *d* orbitals at the neighboring corners are aligned antiphase toward the site outside the hexagon. This is in reminiscence of the alternating phases in the compact localized states of *s*-orbital kagome model in Fig. 1a. In the Supplementary Note 3 and Supplementary Fig. 10, we have demonstrated that such orbital textures combined with fine-tuned multiorbital hopping parameters in the kagome lattice geometry suppress the charge leakage outside the hexagon and localize the real-space electronic wave functions. This finding implies that the destructive quantum phase interference from alternating phases in the *s*-wave kagome tight-binding model is transferred to the real-space chiral orbital textures in realistic *d*-orbital kagome model. This analogy unequivocally confirms the frustration-driven origin of the observed flat bands in CoSn. (3) The Wannier functions centered at the hexagon of the kagome lattice effectively forms a triangular lattice. We estimate the hopping *t* between Wannier functions at neighboring triangular lattice sites to be ≈15 meV reflecting suppressed kinetic energy of the flat band electrons (Supplementary Table 4). In this context, the exotic electronic phases recently derived from the Hubbard model on triangular lattices, including spiral magnetic orders, unconventional superconductivity, and quantum or chiral spin liquids, might be relevant to the flat bands in kagome lattice^44–46^.

### Nontrivial topology of the kagome flat bands

**Fig. 4:**
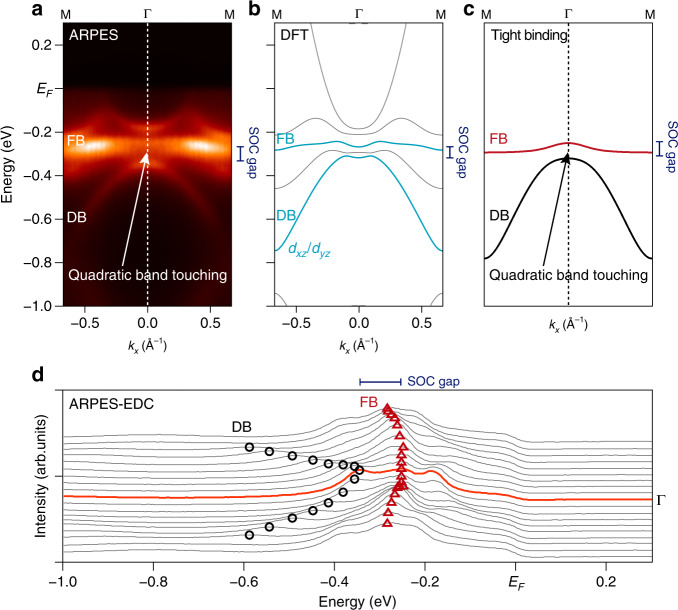
Spin–orbit coupling gap opening between the Dirac and flat bands. **a** High-resolution band structure of CoSn at the vicinity of the Γ point. In this image, we summed up the photoemission intensities from the first, second, and third Brillouin zone to minimize the effect of photoemission matrix element effect in intensity analysis. **b**, **c** Corresponding DFT calculation of CoSn and *s*-wave tight-binding calculation of monolayer kagome lattice for direct comparison with ARPES spectrum in **a**. In **b**, we highlight the Dirac and flat bands (DB and FB) with *d*_*xz*_/*d*_*yz*_ orbital characters with cyan color. **d** Stack of energy distribution curves of the data in **a**. The dispersion of Dirac (flat) band with *d*_*xz*_/*d*_*yz*_ orbital character is tracked with black circles (red triangles). Energy distribution curve at the Γ point (marked with red line) displays the clear separation between the flat and Dirac band peaks, highlighting the gap opening at the quadratic band touching point.

After demonstrating the realization of the kagome-derived flat band in CoSn, we examined the SOC-induced gap opening at the quadratic touching point (at Γ) between the Dirac band and the flat band. In the viewpoint of the prototypical tight-binding framework for the kagome lattice, the gap at the quadratic band touching point is responsible for rendering the flat band topologically nontrivial (see Figs. 1b and 4c), endowing nonzero Chern number/*Z*_2_ invariant under the time-reversal breaking/symmetric condition^10–15^. Figure 4a highlights the band dispersion near the Γ point obtained by averaging three spectra taken at the first, second, and third Brillouin zone to minimize the influence of photoemission matrix element effect to the intensity distributions. The band dispersion closely follows the DFT calculation shown in Fig. 4b, and in particular, it exhibits a quadratic band that emerges from the Dirac band at K (see also Fig. 3c, d) and touches the flat band at Γ. Focusing on this quadratic band and the flat band with same orbital origin (*d*_*xz*_/*d*_*yz*_ orbitals, cyan-colored lines Fig. 4b), the detailed analysis of the energy distribution curves in Fig. 4d clearly reveals the spin–orbit-induced gap opening at the quadratic touching point. The SOC gap size could be quantified to be Δ_ARPES_ = 80 ± 20 meV (Δ_DFT_ = 57 meV), which is considerably larger than the gap observed at the linear band crossing of Fe_3_Sn_2_ ≈30 meV^25^. The direct observation of the SOC gap between the Dirac and the flat band strongly signals the nontrivial topology of the observed flat band at *k*_*z*_ = 0.

**Fig. 5:**
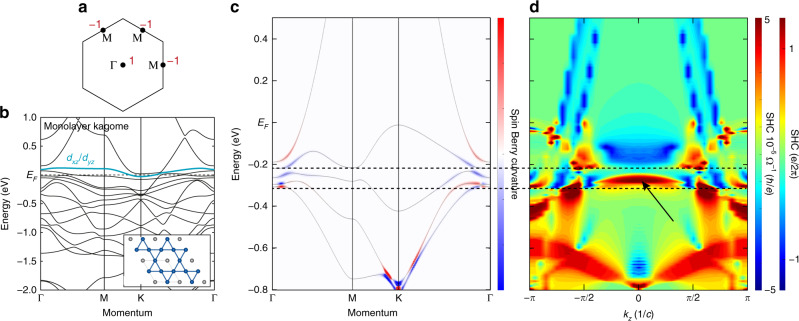
Nontrivial topology of the kagome flat bands in CoSn. **a** Parity eigenvalues of the flat bands of the bulk CoSn at the time-reversal invariant momenta of *k*_*z*_ = 0 plane. **b** Electronic band structure for a single kagome layer limit of CoSn (inset). The *d*_*xz*_/*d*_*yz*_ flat band locates exactly at Fermi level and retains nonzero *Z*_2_ index in the monolayer limit (parity eigenvalues are same with **a**). **c***k*-resolved spin Hall conductivity in the *k*_*z*_ = 0 plane. The dominant contribution to SHC is concentrated near the spin–orbit coupling gap between the flat band and Dirac bands at Γ as well as at the massive Dirac points at Κ. **d***k*_z_ dependence of SHC of CoSn. The in-plane momentum (*k*_*x*_, *k*_*y*_) contribution to the SHC is integrated in this map, up to a given energy. As marked by the black arrow, the SHC is sharply enhanced inside the nontrivial SOC gap between the flat and Dirac bands (horizontal dashed lines). Due to the two-dimensionality of the flat band, its contribution to SHC is fairly *k*_z_ independent across ~40% of the Brillouin zone.

To support this, we use the DFT-derived Wannier tight-binding model to analyze the parity eigenvalue of the flat bands at the *k*_*z*_ = 0 plane following the Fu–Kane formula^47^. This analysis yields a topological index *Z*_2_ = 1 for both flat bands confirming their topological nature (Fig. 5a). We note that the nontrivial topology of the flat bands is also reflected in the spin texture of the constructed Wannier wave functions (Supplementary Fig. 6), where time-reversal symmetry has to be implemented in a nontrivial/nonlocal manner due to the *Z*_2_ obstruction^48^. To exemplify the manifestation of the nontrivial flat band topology on bulk properties, we also calculated the band- and momentum-resolved spin Hall conductivity (SHC) of CoSn (Fig. 5c, d). As displayed in Fig. 5c, the in-plane momentum-resolved SHC is concentrated near the lifted degeneracy point between the Dirac and flat bands, which connects to the topological nature of the latter. The *k*_*z*_-resolved SHC in Fig. 5d additionally demonstrates that, owing to the two-dimensionality of the flat band in CoSn (Figs. 1e and 2i–m), its contribution to the SHC is fairly *k*_*z*_ independent for ~40% of the Brillouin zone, before it hybridizes with other *k*_*z*_-dispersive bands. When extended to the 2D limit of a single kagome sheet, the 2D topological insulator state can be realized based on the topological flat band exactly at the Fermi level (Fig. 5b), where helical edge states and quantized SHC are expected inside the SOC gap. Overall, our experimental observation of the SOC gap between the Dirac and flat bands confirms the realization of another defining properties of 2D kagome band structure in CoSn, which endows nontrivial topological character to the correlated flat band electrons as supported by our calculations.

In sum, we have successfully discovered and characterized the topological flat bands in the frustrated kagome lattice CoSn. An important future research direction is to raise the kagome flat band to the Fermi level to observe the proposed lattice-borne correlated topological phases. Potential routes to this include bulk doping (e.g., by Co_1 − *x*_Fe_*x*_Sn, ref. ^49^), monolayer fabrication, and application of compressive in-plane strain as suggested by our DFT calculations (Supplementary Figs. 7–9). Altogether, the observation of topological flat bands in the kagome lattice opens up a new avenue to study correlation-driven emergent electronic phenomena on the background of topological nontriviality.

## Methods

### Synthesis of CoSn single crystals

Single crystals of CoSn were synthesized using an Sn self-flux method. Cobalt powder (Alfa Aesar, 99.998%) and tin pieces (Alfa Aesar, 99.9999%) were mixed with a molar ratio of 1:9 and loaded in an alumina crucible and sealed in a quartz tube under high vacuum. The tube was heated to 950 °C and maintained for 5 h, and was gradually cooled to 650 °C with a typical cooling rate of 2–3 °C/h. At 650 °C the tube was removed from the furnace and centrifuge dissociation was performed to separate the crystals from flux. Crystals with a hexagonal prismatic shape were obtained with the longest dimension (6–8 mm) typically along [001].

### Angle-resolved photoemission spectroscopy

ARPES experiments were performed at Beamline 4 (MERLIN) and Beamline 7 (MAESTRO) of the Advanced Light Source equipped with R8000 and R4000 hemispherical electron analyzers (Scienta Omicron), respectively. For ARPES measurements, surface of CoSn was prepared in two different ways, one by ex situ fine polishing followed by in situ Ar^+^ ion sputtering and annealing at 1000 °C and the other by in situ low-temperature cleaving. The experiments were performed below ≈60 K and under the ultrahigh vacuum ≈4 × 10^−11^ torr. The photon energy was scanned in the range from 30 to 160 eV, which covers more than two full three-dimensional Brillouin zone of CoSn. Corresponding *k*_*z*_ momentum was calculated by assuming nearly free-electron final state with inner potential 5.5 eV. We used *s*-polarized photons unless specified, which maximize the signal from the flat bands. The convoluted energy and momentum resolutions of the beamline and the analyzer were better than 30 meV and 0.01 Å^−1^, respectively. Termination dependence or the effect of surface polarity have not been observed in our ARPES experiment.

### Electronic structure calculation from the first principles

DFT calculations were performed with the Vienna Ab initio Simulation Package^50,51^. The pseudo-potentials are of the Projector Augmented Wave^52^ type with exchange-correlation energy functional parameterized by Perdew, Burke, and Ernzerhof^53^ within the generalized gradient approximation. The bulk DFT calculations are converged with plane-wave energy cut-off 350 eV and a reciprocal space Monkhorst–Pack grid sampling of size 15 × 15 × 11. The SOC terms are included for the electronic band structure. The calculation of the effective interaction parameters *U* in CoSn is performed based on the linear response approach following ref. ^38^ (see the Supplementary Fig. 14 for details). We note that the theoretical Fermi level from the DFT needs to be shifted down by 140 meV to fit the experimental band structure, presumably due to a slight Sn off-stoichiometry in our crystals. Such shifting has been applied in the calculations presented in Figs. 1e, 2h, 3b, d, 4b, and 5c, d, and Supplementary Figs. 3, 5, and 13.

### Wannier tight-binding model of CoSn

To interpret the first-principle calculations, the post-process Wannier90 code^54^ is used to convert the extended periodic Bloch wave function basis in the DFT into the localized Wannier functions basis in the real space^55^. With this Wannier transformation, the effective tight-binding Hamiltonian for a selected group of bands of the material can be constructed. The construction gives both the accurate DFT bands and physically transparent pictures of localized atomic orbitals and their mutual hybridizations. Here, we have included Co 3*d* states, and Sn 5*s* and 5*p* states in the Wannier construction for the bands around the Fermi level. The Wannier model is first constructed without SOC. The SOC interactions are modeled with atomic terms, which capture the essential feature of the DFT bands with SOC. With these Wannier models, the symmetry properties and the topological index could be directly computed. In the Supplementary Fig. 5a, b, we show the band structure comparison between the full DFT calculations and the interpolated Wannier tight-binding model with and without SOC modeling, respectively. The extracted SOC strength $\lambda\vec{L}\cdot\vec{S}$ has *λ*_Co_ ≈ 70 meV for Co atoms and *λ*_Sn_ ≈ 290 meV for Sn atoms. Hybridization between Co and Sn orbitals may account for the larger mass of the Dirac electrons at K (see Supplementary Figs. 3a and  5b) compared to related kagome compounds Fe_3_Sn_2_ and FeSn^25,26^. The constructed Wannier tight-binding model for CoSn allows us to perform ab initio calculations for the SHC and the *k*-resolved contributions from each band, using the Kubo formula following refs. ^56,57^.

## Supplementary information

Supplementary Information

Peer Review File

## Data Availability

The data that support the plots within this paper and other findings of this study are available from the corresponding author upon reasonable request.
