# Supplementary material for: Topological flat bands in frustrated kagome lattice CoSn
Source: Nat Commun. 2020 Aug 10;11:4004. doi: 10.1038/s41467-020-17465-1 (PMC7417556; doi:10.1038/s41467-020-17465-1)
Supplement: Supplementary file 1 — Supplementary Information [file 41467_2020_17465_MOESM1_ESM.pdf]

Supplementary Information

Topological flat bands in frustrated kagome lattice CoSn

Mingu Kang et al.,

Supplementary Figures 1-14

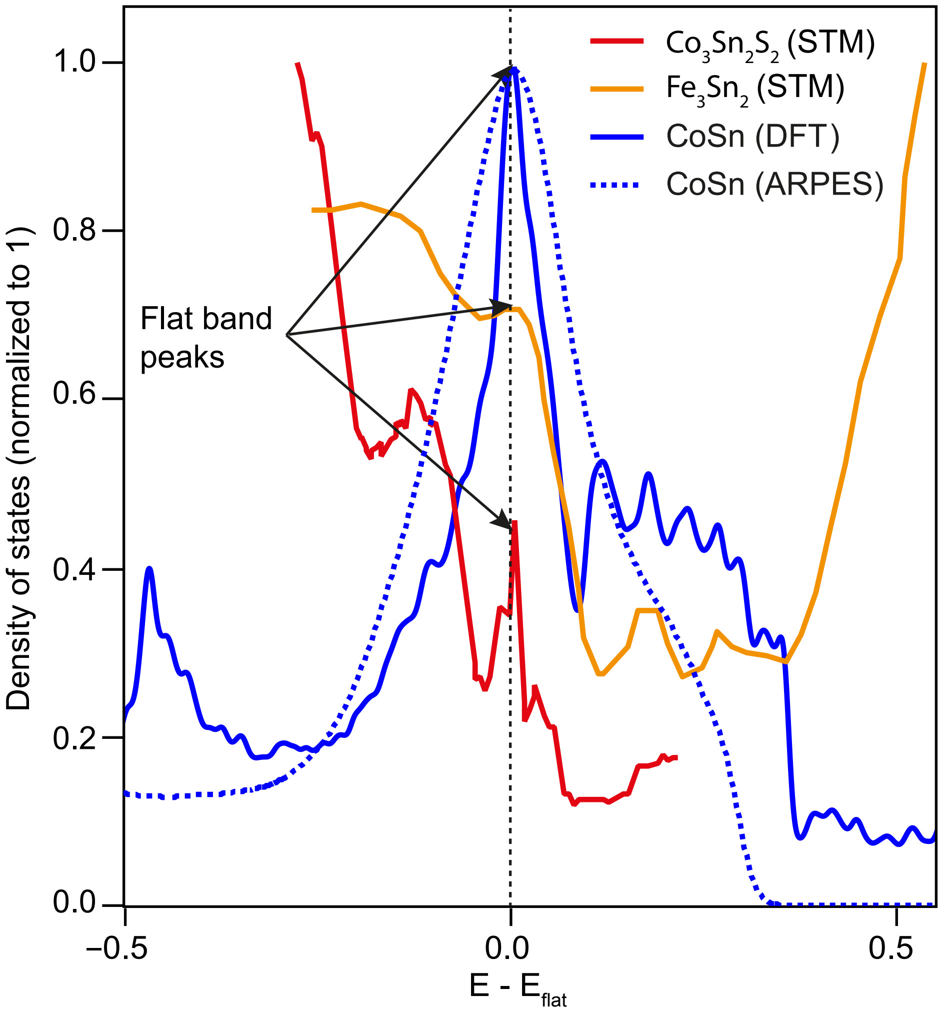

Supplementary Figures 1 | Comparison between putative flat band density-of-states in $Co_3Sn_2S_2$, Fe_3Sn_2, and CoSn. The STM data of $Co_3Sn_2S_2$ are reproduced from Ref. 10, which exhibits same flat band feature with the data in Ref. 11, but has more extended energy range. The Fe_3Sn_2 STM data are reproduced from Ref. 12. In both $Co_3Sn_2S_2$ and Fe_3Sn_2, the DOS enhancements from the claimed 'flat' bands are actually marginal compared to the overall DOS, suggesting a rather limited momentum-space range of flat dispersion. In contrast, the flat band in CoSn clearly dominates the DOS in both DFT calculation and ARPES experiment, as expected from the ideal flat band.

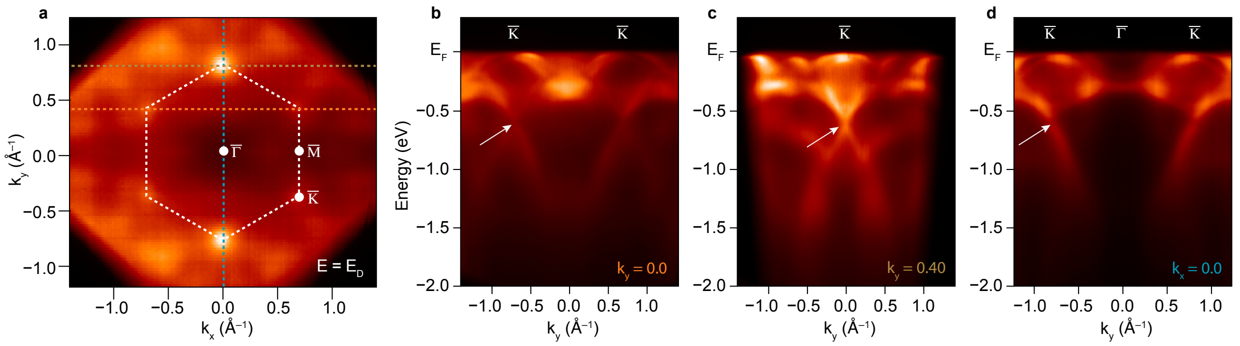

Supplementary Figures 2 | Dirac fermions in CoSn. a, Constant energy map of CoSn at the Dirac point energy ($E_D = -0.57$ eV). Dirac points at $\overline{K}$ of the hexagonal surface Brillouin zone can be observed. **b-d,** Energy-momentum dispersion of CoSn across $\overline{K}$ points measured along various momentum space directions: at $k_y = 0.40$ $Å^{-1}$ (b), $k_y = 0.79$ $Å^{-1}$ (c), and $k_x = 0.0^{\circ}$ $Å^{-1}$ (d). Linear crossing at the Dirac points (marked with white arrows) can be observed in all momentum space directions confirming the realization of Dirac fermions in CoSn. We quantified the Dirac velocity as $v_D = 1.8 \times 10^6$ m/s, which is one order of magnitude smaller than that of graphene and comparable to those observed in related magnetic kagome metals Fe_3Sn_2 and FeSn.[6,7] All data were measured with *s*-polarized 97 eV photons, which correspond to $k_z = \pi$ (mod 2π) according to our photon energy-dependent ARPES experiment.

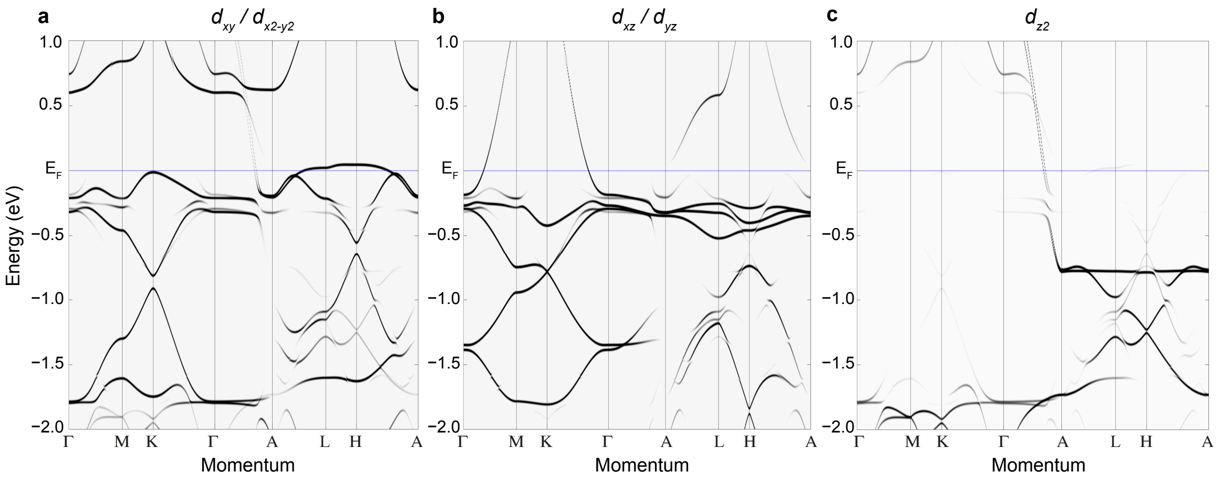

Supplementary Figures 3 | Orbital decomposition of the DFT band structure of CoSn. a-c, Orbital weights of d_{xy}/d_{x2-y2}, d_{xz}/d_{yz}, and d_{z2} orbitals on the DFT bands of CoSn respectively.

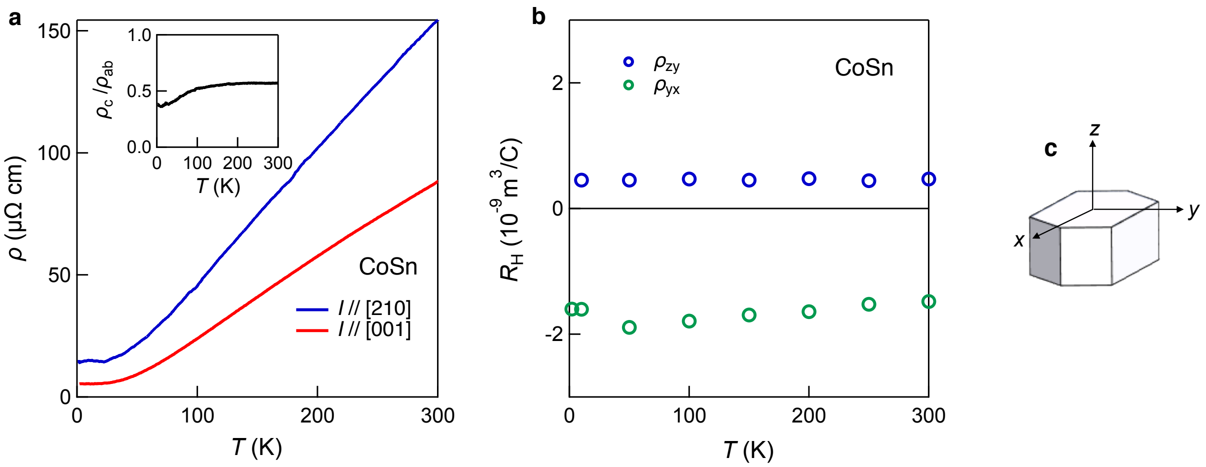

Supplementary Figures 4 | Transport characterization of CoSn single crystals. a, The resistivity as a function of temperature with current flown along [210] (blue curve) and [001] (red curve) directions. Inset shows the ratio between out of plane and in plane resistivities. **b,** Hall coefficient measured with in-plane (green circles) and out-of-plane (blue circles) configurations. **c,** Schematic of the Cartesian coordinates with respect to the crystallographic axis.

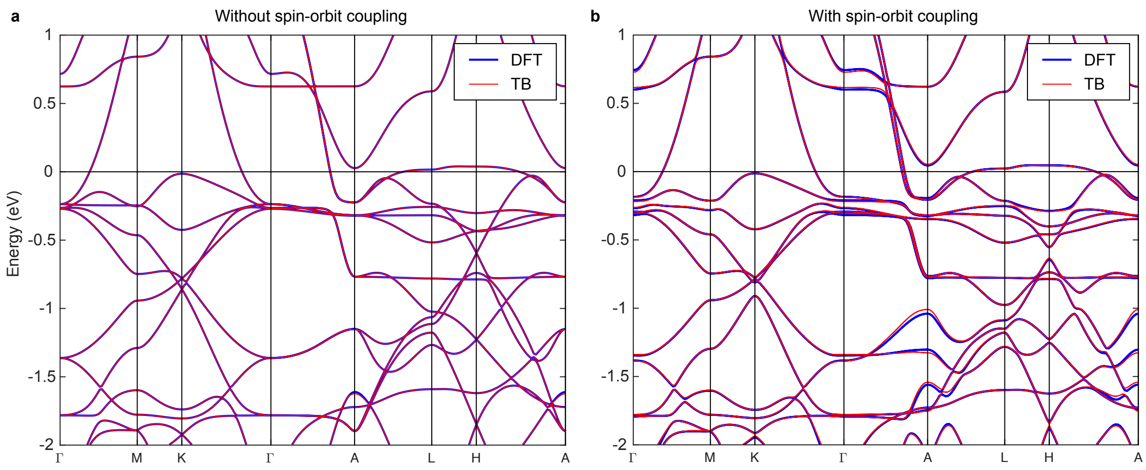

Supplementary Figures 5 | Comparison between the DFT calculation and the Wannier tight-binding model of CoSn. a,b, Band structure of CoSn obtained from DFT (blue) and the Wannier tight-binding model (red) with and without spin-orbit coupling respectively. The spin-orbit coupling is included as an atomic term $\lambda\vec{L}\cdot\vec{S}$.

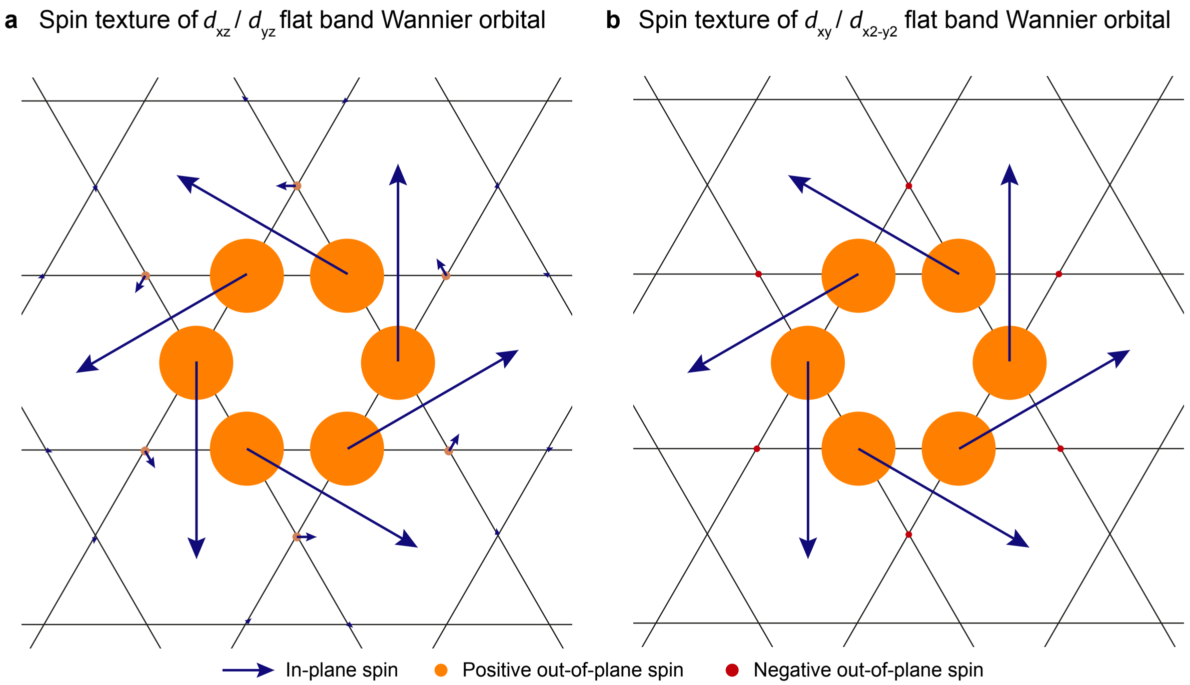

Supplementary Figures 6 | Real-space spin texture of the flat band Wannier functions. a,b, Spin-texture of the d_{xz}/d_{yz} and d_{xy}/d_{x2-y2} flat band Wannier functions respectively. In-plane spin directions are marked with blue arrows, while the out-of-plane spin directions are marked with orange and red circles. The length scales of arrows and circles are proportional to the magnitude of spin expectation values on each site.

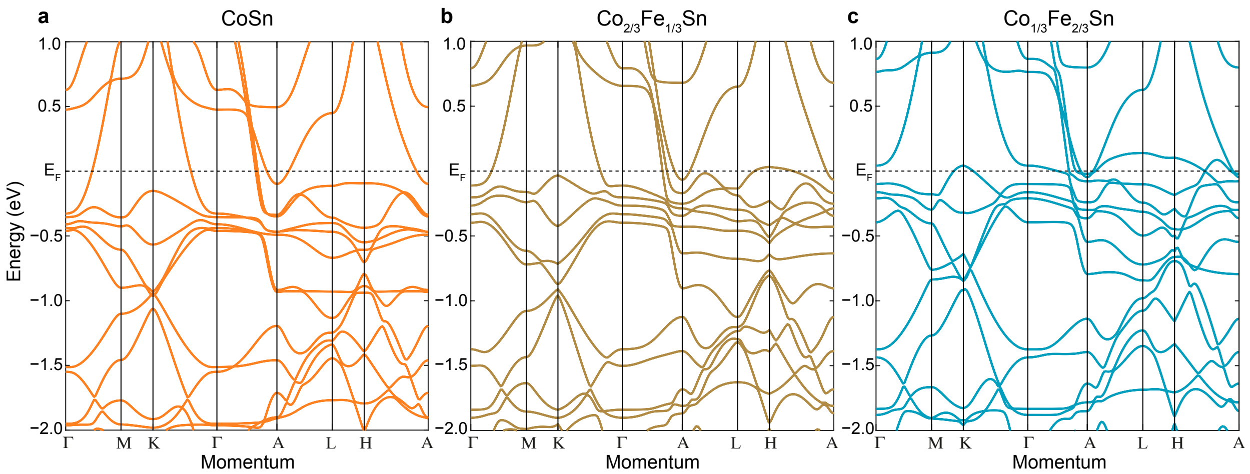

Supplementary Figures 7 | Tuning the flat band energy of CoSn via bulk Fe doping. a-c, DFT band structure of $Co_{1-x}Fe_xSn$ with x = 0, 1/3, and 2/3 respectively. The d_{xy}/d_{x2-y2} derived flat band at k_z = 0 crosses the Fermi level at x = 2/3 doping, while more electrons are required to position the d_{xz}/d_{yz} at the Fermi level. Nonmagnetic ground states are assumed in all cases.

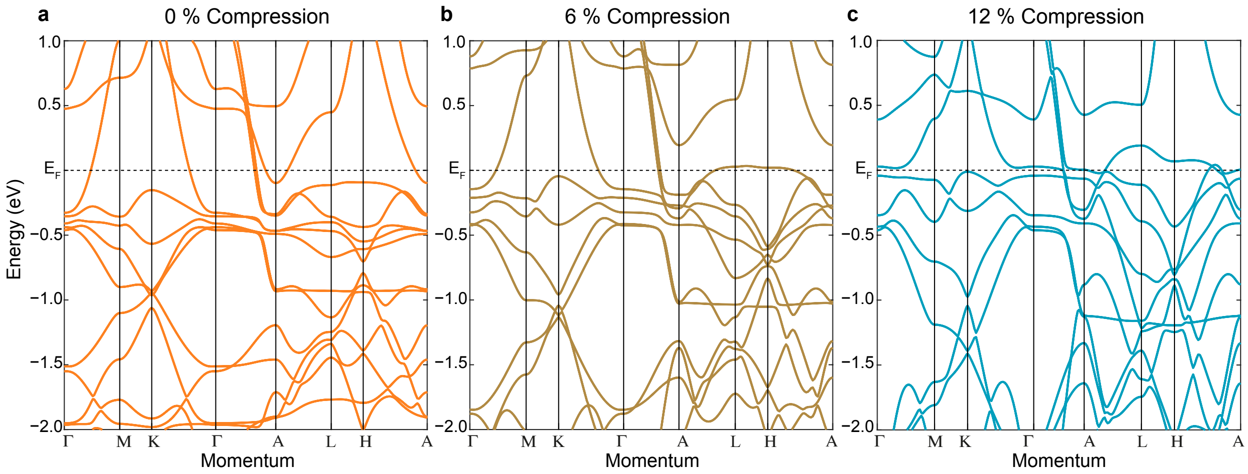

Supplementary Figures 8 | Tuning the flat band energy of CoSn via in-plane compressive strain. a-c, DFT band structure of CoSn with in-plane compressive strain of 0 %, 6 %, and 12 % respectively. Such in-plane compressive strain mimics/simulates the case of CoSn thin film grown on lattice-mismatched substrate. The out-of-plane lattice constant is relaxed on each calculation. The flat band moves upward with the in-plane compressive strain, and can be almost aligned to the Fermi level with 12 % compression.

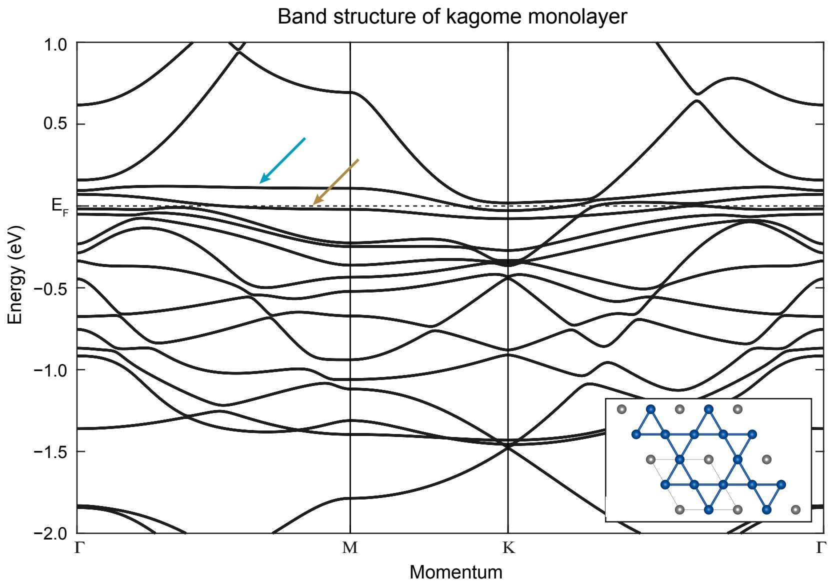

Supplementary Figures 9 | Tuning the flat band energy of CoSn via monolayer fabrication. DFT band structure of monolayer kagome lattice consists of Co and Sn as shown in the inset. In the monolayer limit, flat bands from d_{xz}/d_{yz} and d_{z2} orbitals locate at the very vicinity of the Fermi level or cross it as marked with cyan and brown arrows respectively. The shift of flat band energy is partly due to the absence of the spacer Sn layer in the monolayer kagome limit, from which electrons are donated to the kagome layer in bulk CoSn. Our analysis of the parity eigenvalues of the flat bands in monolayer limit reveal their non-trivial topology similar to the case of $k_z = 0$ flat bands in bulk CoSn.

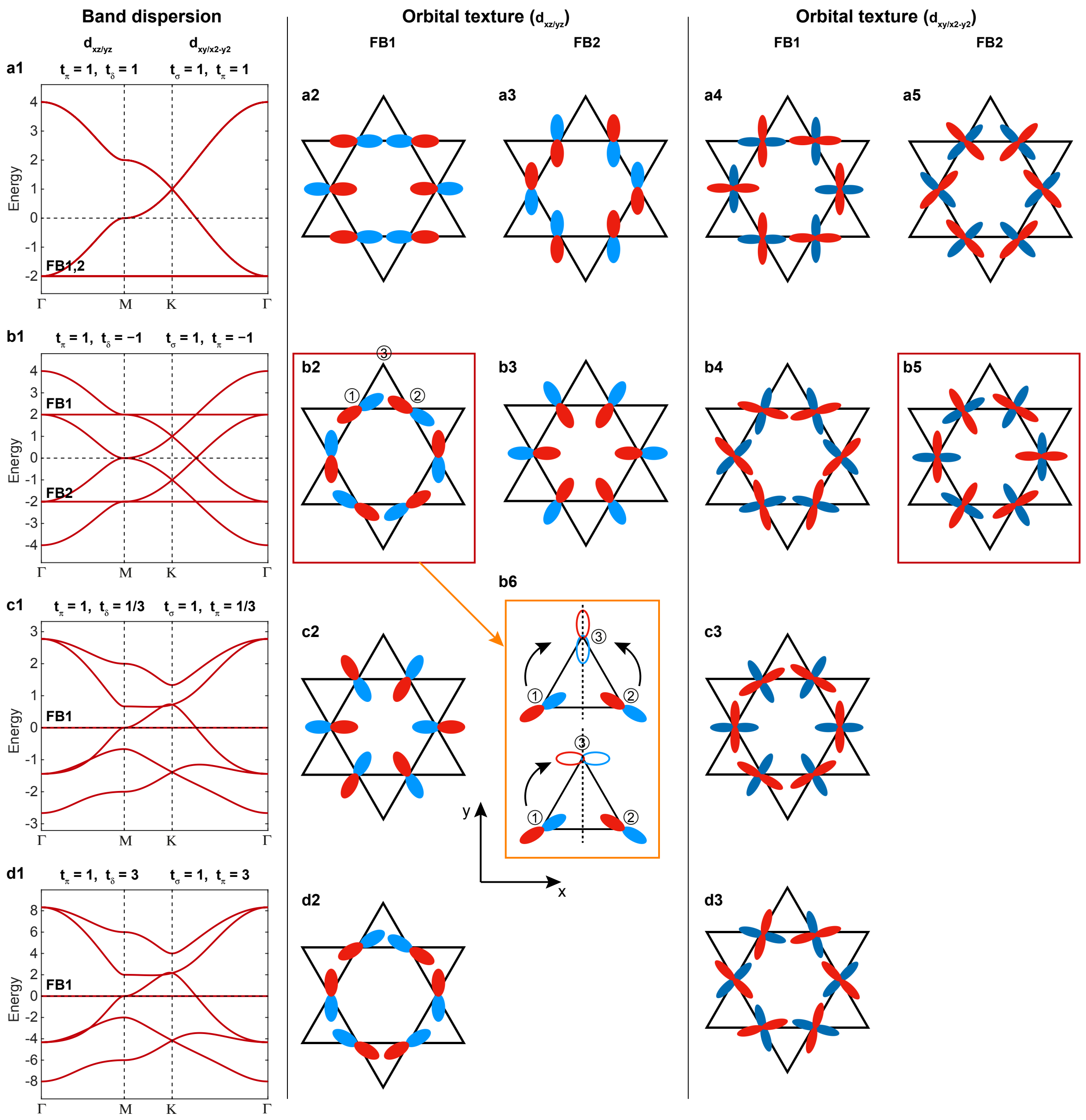

Supplementary Figures 10 | *d*-orbital tight-binding calculations of kagome lattice and the real-space orbital textures of the flat band eigenstates. Despite the complexity arising from the multiple orbital hopping channels the flat band manifests at specific t_π/t_δ or t_σ/t_π. The orbital textures in b2 and b5 resemble those of flat bands in CoSn.

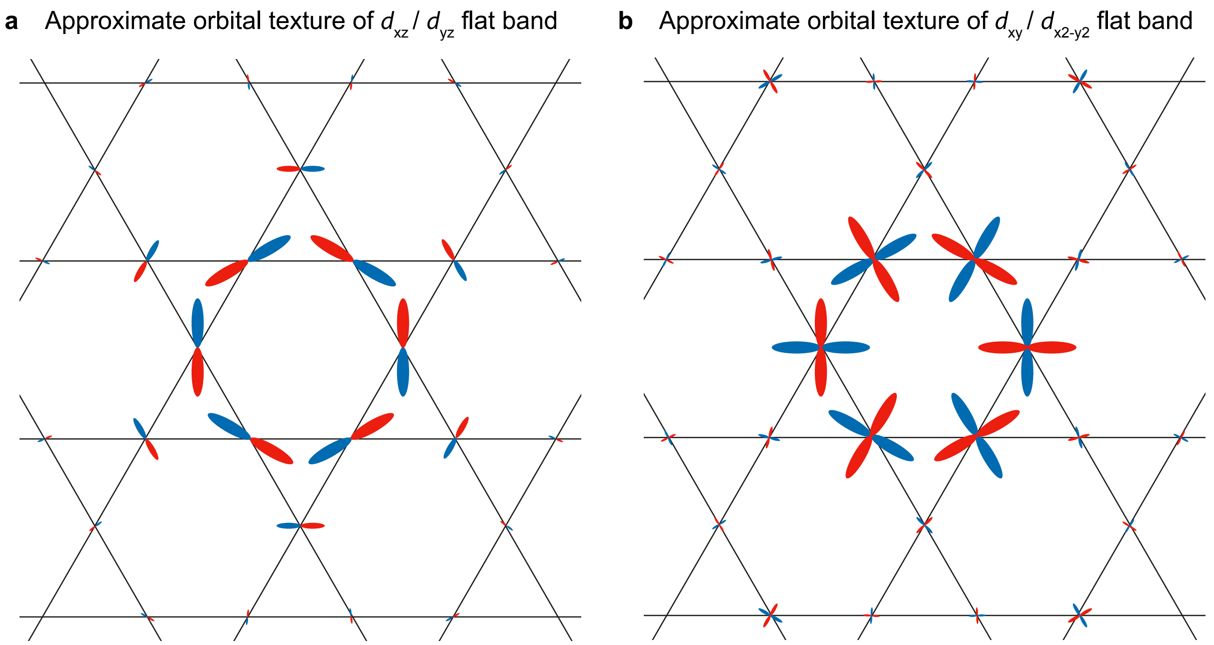

Supplementary Figures 11 | Approximate orbital textures of the flat bands in CoSn derived from the spinless model. The length-scale of depicted orbitals are proportional to the magnitude of the wave function at each lattice site.

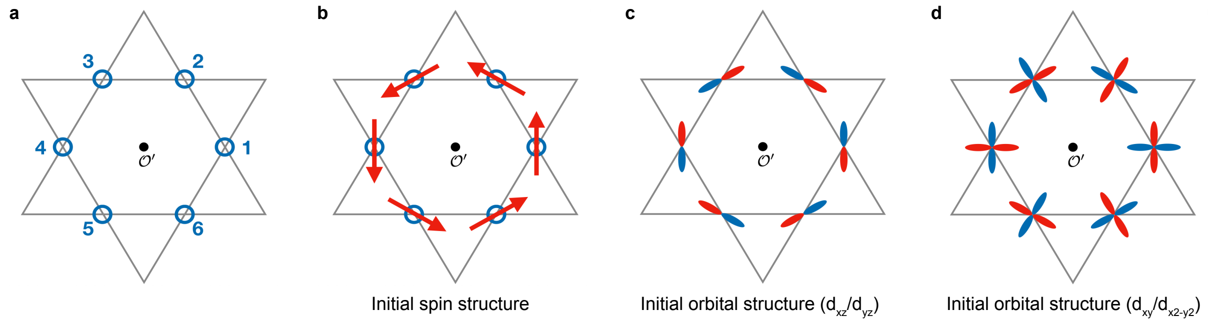

Supplementary Figures 12 | Initial seeding function for Wannier projection. a, The initial Wannier seeding function has non-zero wavefunction on the six sites ($i = 1$ to $i = 6$) of the hexagon corners. **b,** The spin texture of the seeding function. **c,d,** The orbital texture of the seeding function in projecting d_{xz}/d_{yz} and d_{xy}/d_{x2-y2} flat bands.

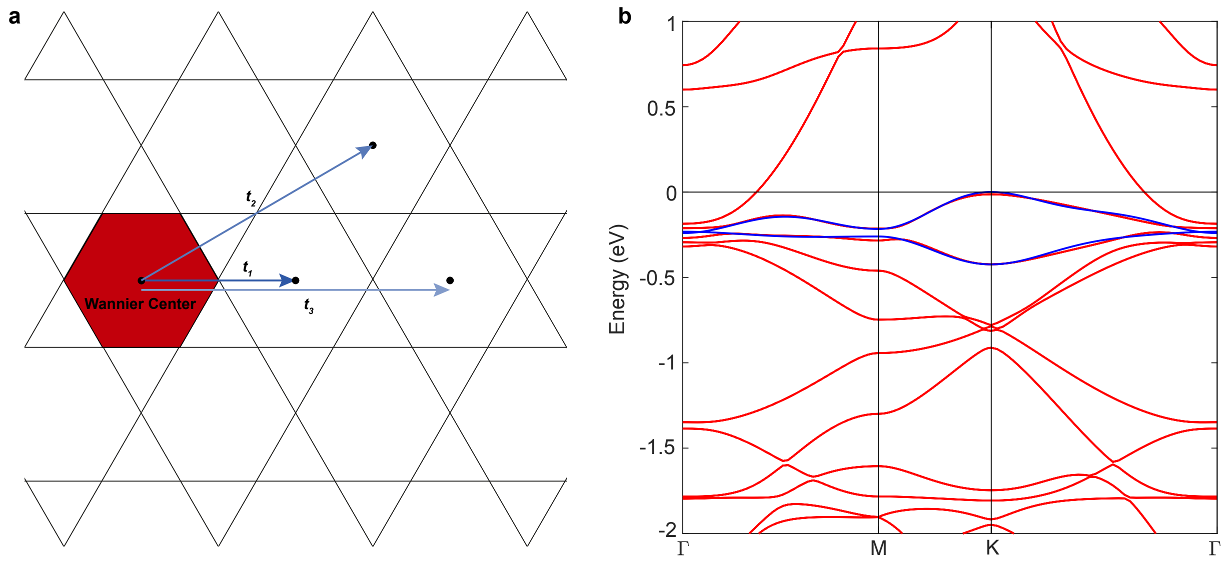

Supplementary Figures 13 | Effective Wannier tight-binding model for the flat bands in CoSn. **a,** Schematics of the first, second, and third nearest neighbor hoppings between the flat band Wannier functions centered at each hexagon of kagome lattice. **b,** Band structure of the effective flat band Wannier tight-binding model (blue lines). The hopping parameters resulting from the fit to the DFT band structure (red lines) at $k_z = 0$ are given in the Supplementary Table 4.

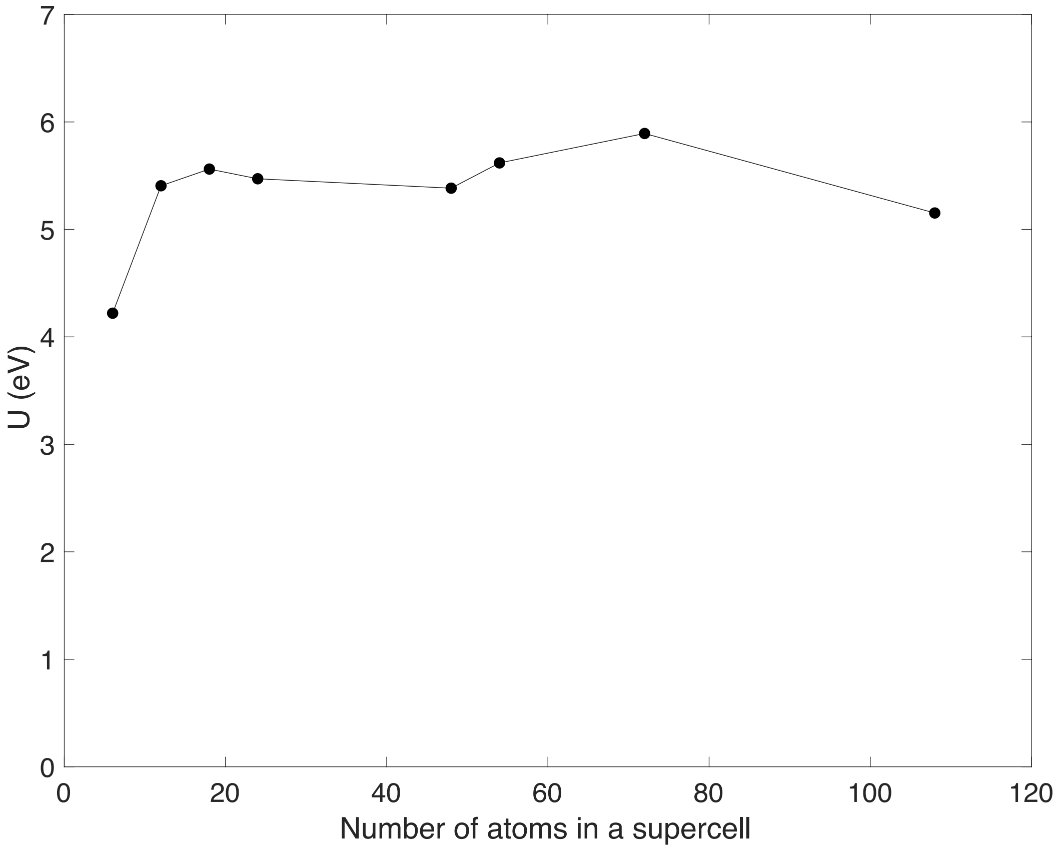

Supplementary Figures 14 | Calculation of the effective interaction parameter *U* for Co atom in CoSn based on the Linear response approach. The calculation is performed with varying supercell sizes 1*1*1, 1*1*2, 1*1*3, 2*2*1, 2*2*2, 3*3*1, 2*2*3, and 3*3*2. The corresponding number of atoms in the supercells is 6, 12, 18, 24, 48, 54, 72, and 108 respectively. The convergence of *U* is nonmonotonic with respect to the number of atoms due to the different aspect ratios in each supercell. However, the resulting *U* values always fall in 5~6 eV range except that from the smallest 1*1*1 calculation.

Supplementary Note 1. Transport characterization of CoSn single crystals

We have characterized the transport properties of CoSn single crystals using the standard five probe technique with AC current excitation in a commercial superconducting cryostat. The crystals show metallicity (Supplementary Figure 4a) along both in and out-of-plane directions with residual resistivity ratio between 11 and 16. The steep k_z Fermi velocity associated with the d_{z2} bands (see Fig. 1e) may account for the lower out-of-plane resistivity observed in Supplementary Figure 4a.

The Hall coefficients shown in Supplementary Figure 4b are estimated from the slope of the Hall resistivity between ±1 T and are weakly temperature dependent for both the in and out of plane configurations (Supplementary Figure 4c). The negative in-plane Hall coefficient is consistent with the ARPES observation and DFT calculation of a large electron pocket centered at Γ. The positive out-of-plane Hall coefficient, implying an overall hole behavior, may be related to the Fermi surfaces originated from d_{z2} orbitals.

Supplementary Note 2. Tunning the flat band toward the Fermi level

To realize exotic correlated electronic phases driven by flat band electrons, the flat band should be located at the Fermi level. The binding energy (0.2~0.3 eV) of the observed flat bands in CoSn is already close to the Fermi level, and in Supplementary Figure 7-9, we propose various DFT-guided pathways to further tune the flat band position toward the Fermi level. The proposed methodology includes bulk doing (Supplementary Figure 7), compressive strain (Supplementary Figure 8), and reducing dimensionality (Supplementary Figure 9).

Supplementary Note 3. Wannier localized states of the *d*-orbital flat bands

In a kagome lattice decorated with *s*-wave orbitals, the flat bands can be understood from the localized states as shown in Fig. 1a. In this localized state, the electrons reside on the six corners of the hexagon. This state is an exact energy eigenstate for a kagome model with only nearest-neighbor hopping. The eigenstate and localization properties can be traced back to the wave function phase interference.

We note here about the physical properties of these hexagon localized states. Two adjacent hexagon states are not orthogonal with each other due to the wave function overlap at the shared kagome site. In a torus geometry of kagome lattice with N hexagon site, only N-1 hexagon states are independent (since when the kagome lattice is occupied with these hexagon states at all hexagon centers, the superposition of them gives rise to a null wave function). Another equivalent view is that, these hexagon states are not well-defined at $\vec{k} = 0$ in the momentum space. Apart from such hexagon states, there are additional non-contractible loop states that can emerge in a torus geometry.[1] By counting the independent hexagon states and loop states, one can infer the features in the bulk electronic spectrum such as the eigenstate degeneracies.[1]

An alternative way to derive these localized hexagon states is from the superposition of flat band eigenstates in the momentum space. We know this cannot be a linear superposition with uniform weight in the momentum space due to the singularity as $\vec{k} = 0$, which is doubly degenerate. A proper weighting function and gauge choice in the momentum $\vec{k}$ that excludes the states at $\vec{k} = 0$ has been analytically derived in case of the *s*-wave kagome lattice to generate these exact hexagon states.[1]

Although these hexagon states are intuitive objects in gaining insights into the wavefunction texture of the flat bands, these objects themselves are not proper orthonormal real-space Wannier function basis. Later we will see how the spin-orbit coupling gives rise to isolated flat band manifold (removing the $\vec{k} = 0$ double degeneracy) and promote such hexagon states into orthonormal Wannier states by introducing intricate spin textures and orbital spin entanglement.

In CoSn material, the kagome sites are decorated by the *d*-orbitals which can be more complicated than the isotropic *s*-wave orbitals discussed above. We can classify these *d*-orbital states into three groups, the d_{z2}, d_{xz}/d_{yz} and d_{xy}/d_{x2-y2} by their angular momentum L_z. Based on the comparison of ARPES and DFT presented in the main text, our main interest is on the derivation

of Wannier objects or compact localized states associated with the d_{xz}/d_{yz} and d_{xy}/d_{x2-y2} flat bands. The d_{z2} group resembles the *s*-orbitals, and the analogy can be made for their electronic structures (Though there exists a realistic limitation in d_{z2} orbital-driven flat band that their out-of-plane orbital orientation enhances the k_z band dispersion as discussed in the main text).

Below, we present three different methods to motivate and derive Wannier(-like) localized states associated with *d*-orbital based flat bands. First, in the subsection A and B, we considered *d*-orbital-based kagome tight-binding model with perfectly flat bands as the simplest case. From this, we could directly construct the real-space localized states possessing characteristic orbital textures. The localized states in these cases are fully confined in the single hexagon of the kagome lattice similar to the *s*-wave kagome model in Fig. 1a. Second, in the subsection C, we considered more realistic flat bands of CoSn with small but finite band dispersion. We first investigated spinless case (i.e. without inclusion of the spin-orbit coupling) to avoid complication from the spin-orbital entanglement. As the first-order approximation, we adopted analytically known *k*-space weighting function of the *s*-orbital kagome model[1] to construct the corresponding localized states. The resulting orbital textures are similar to those derived from the first method, though there appears small but finite electron density outside of the hexagon due to the nonzero dispersion of the flat bands in CoSn. Finally, in the subsection D and E, we considered the most realistic spinful case of the CoSn flat bands. Spin-orbit coupling lifts the degeneracy at $\vec{k} = 0$ and isolate the flat bands from other bands, and the flat band Wannier functions could be rigorously constructed. The resulting spin-orbit entangled Wannier states could be locally approximated as a product of (majority) spin and orbital wave functions using Schmidt decomposition. In all three cases, the orbital textures of the constructed localized states are qualitatively similar as shown in Supplementary Figure 10, Supplementary Figure 11, and Fig. 3e,f, confirming the validity of our approaches and approximations used.

A. Spinless d_{xz}/d_{yz} kagome lattice.

First, we consider a kagome lattice with sites decorated by the spinless d_{xz}/d_{yz} orbitals. When projected on the *xy* plane, this set of orbitals resemble the *p*-wave p_x and p_y orbitals. For simplicity, we assume only nearest neighbor coupling between orbitals. In a rotated new frame with the closest neighbor along the $\hat{x}$ axis, the hopping strength between d_{xz} (d_{yz}) orbitals is t_π (t_δ) with vanishing crossing terms due to (approximate) *xz* mirror.

At specific t_π/t_δ values (i.e. t_π/t_δ = 1, -1, 1/3, 3), we find perfect flat bands emerge in the electronic structure spectrum as illustrated in Supplementary Figure 10. Similar to the *s*-wave kagome lattice, the perfect localized states confined to the hexagon could be constructed by considering the destructive interference in the Hamiltonian. The d_{xz}/d_{yz} orbital texture of the localized states are depicted in Supplementary Figure 10 for various t_π/t_δ values.

B. Spinless d_{xy}/d_{x2-y2} kagome lattice.

Similar analysis as above can be carried out for a kagome lattice decorated with d_{xy} and d_{x2-y2} orbitals on each site. In a rotated new frame with the nearest neighbor site along $\hat{x}$ axis, the coupling between d_{x2-y2} (d_{xy}) orbitals is t_σ (t_π). Crossing terms are also assumed to vanish due to (approximate) *xz* mirror plane. Specific t_σ/t_π ratios (i.e. t_σ/t_π = 1, -1, 1/3, 3) are identified with perfect flat bands in the electronic structure. The band structure and the orbital texture of the associated localized states are again illustrated in Supplementary Figure 10.

In Supplementary Figure 10b6, we illustrate the importance of the derived orbital textures in the destructive interference of hoppings on *d*-orbital kagome lattice. Here we considered the orbital textures in Supplementary Figure 10b2 as an example, but the description can be easily extended to other orbital textures. An important difference with *s*-orbital case is the complexity arising from the multiple orbital hopping channels, i.e. hoppings to outside of the central hexagon can occur to two orbitals (d_{xz}/d_{yz}) in the receiver site (sublattice 3 in the figure). If we define *yz* mirror plane crossing the sublattice 3, the *d*-orbitals at the sublattice 3 are either even (d_{yz}) or odd (d_{xz}). In the former case, the hoppings from sublattices at the central hexagon (sublattice 1 and 2) are cancelled with each other as the *d*-orbitals at sublattices 1 and 2 are aligned antiphase toward the sublattice 3. This mechanism is analogous to the destructive interference from alternating phases in the *s*-orbital kagome model. In case of hoppings to odd orbital at sublattice 3 however, the same argument results in the constructive interference between hoppings from sublattice 1 and 2, and an additional mechanism is required to suppress the delocalization of the wave function. Indeed, in the given orbital textures, the overlap integral between site 1 and 3 (or between site 2 and 3) are tuned to zero at the specific t_π/t_δ ratios (in this case $t_\pi/t_\delta = -1$). This finding indicates that the characteristic orbital texture of localized states combined with fine-tuned hopping parameters can give rise to the flat band in *d*-orbital kagome lattices.

C. Spinless CoSn flat bands – the first order approximation.

We now move on to the more realistic (quasi-)flat bands at $k_z = 0$ of the CoSn band structure. We first considered spinless bands to gain some intuitions about the orbital textures. The electronic structure without spin-orbit coupling is shown in Supplementary Figure 5a. Based on the *xy* mirror symmetry, the bands can be classified into odd and even symmetry sectors. Under this symmetry classification, two sets of flat bands (one odd type d_{xz}/d_{yz} and the other even type d_{xy}/d_{x2-y2}) can be easily identified from other bands apart from the degeneracies at Γ point.

To construct the localized states from the identified flat band manifold of both sectors, we employed the weighting function used previously in the *s*-wave kagome lattice (as the first approximation) to sample the flat band eigenstates over the Brillouin zone. The ideal localized states found above in various limits of the model are used as the trial states to define the gauge condition in taking the weighted superposition. To be specific, we have the flat band eigenstate $|\psi^{(f)}(\vec{k})\rangle$ and trial state $|\phi(\vec{k})\rangle$ from the ideal localized states at momentum $\vec{k}$. The phase of $|\psi^{(f)}(\vec{k})\rangle$ is fixed by requiring real and positive $\langle\phi(\vec{k})|\psi^{(f)}(\vec{k})\rangle$ (i.e. gauge condition). One should also monitor the singular $\vec{k}$ points where $\langle\phi(\vec{k})|\psi^{(f)}(\vec{k})\rangle = 0$ apart from $\vec{k} = 0$ point. If it is non-singular for every non-zero $\vec{k}$, we can perform the superposition to obtain the localized real-space state

$$|\phi'(\vec{R})\rangle = N \int d^2\vec{k} e^{-i\vec{k}\cdot\vec{R}} \omega(\vec{k}) |\psi^{(f)}(\vec{k})\rangle \quad (1)$$

with *N* the normalization factor, and the weighting function $\omega(\vec{k}) = \sqrt{\sum_{\nu=1}^{3} \sin^2(\vec{k}\cdot\delta_\nu)}$ adopted from the *s*-wave flat band model[1] where δ_ν are the three nearest neighbor hopping vectors. The charge densities and orbital textures of the resulting localized functions are shown in Supplementary Figure 11. Despite of the leakage of charge densities outside of the central hexagon, the orbital textures around the hexagon resemble those derived in the section A,B (Supplementary Figure 10b2,b5). This suggests that the bandwidths of the flat bands in CoSn are suppressed by the same mechanism discussed above.

D. CoSn flat bands with spin-orbit coupling: Wannier construction

With inclusion of the spin-orbit coupling, two sets of flat bands at the $k_z = 0$ plane are isolated from the rest of the bands. The goal is to construct an effective tight-binding model description in each set of the flat bands, in terms of spatially localized Wannier function basis.[2] First, we would like to investigate if it is possible to derive such localized exact Wannier states from the given band manifold in CoSn, and under what conditions.

Due to the nontrivial band topology, it is impossible to derive fully symmetric Wannier functions for the pair of flat bands.[3] Such Wannier obstructions can be understood as following: Suppose the Wannier functions represent a symmetry g naturally. Then by rotating to the Wannier basis one can describe the energy bands using a g-symmetric tight-binding model. The resulting tight-binding model consists of nothing but the bands of interest, and therefore the band topology of the bands must be trivial. This would lead to a contradiction if the original bands have nontrivial band topology protected by g.

From the preceding discussion, we see that, in order to derive Wannier functions for the pair of nearly flat bands, we have to first identify the symmetries which protect the nontrivial band topology. Due to the nontrivial Z_2 invariant, time-reversal symmetry cannot be manifest in the Wannier functions.[3] Furthermore, due to the xy mirror symmetry, the bands can also be characterized by their mirror Chern number. The nontrivial Z_2 invariant implies the mirror Chern number is odd, and therefore one must also forgo the xy mirror symmetry in the Wannier functions. Finally, the inversion symmetry, which is utilized in the Fu-Kane parity criterion[4] to diagnose the Z_2 invariant, also leads to a more subtle form of Wannier obstruction.[5] With all these symmetries forgone, the Wannier functions can at most be manifestly symmetric under the C_6 rotation about its charge center, together with the combination of inversion and perpendicular mirrors with the time-reversal symmetry, as discussed in the main text.

The complementary symmetry aspects can be viewed from the momentum space description. At high symmetry points, one can analyze the symmetry characters of the group of bands as in Supplementary Table 1 for the flat bands. The proposed Wannier states with specific symmetry representations and Wannier centers have to reproduce the same set of symmetry representations at these high symmetry points.

	Γ	M	K
P	(1,1)	(−1, −1)	-
C_{6z} (d_{xz}/d_{yz})	$e^{\pm i\pi/6}$	-	-
C_{6z} ($d_{xy}/d_{x2\text{-}y2}$)	$-e^{\pm i\pi/6}$	-	-
C_{3z}	$e^{\pm i\pi/3}$	-	$e^{\pm i\pi/3}$
M_{xy}	$\pm i$	$\pm i$	$\pm i$
M_{xz}	$\pm i$	$\pm i$	$\pm i$
My_z	$\pm i$	$\pm i$	-
C_{2x}	$\pm i$	$\pm i$	$\pm i$
C_{2y}	$\pm i$	$\pm i$	-

Supplementary Table 1. The symmetry representations of the d_{xz}/d_{yz} or d_{xy}/d_{x2-y2} flat bands. The values indicate the symmetry eigenvalues under the symmetry operations.

With the symmetry representation and orbital texture of the localized states projected from the spinless model, we used the initial seeding functions for the Wannier projection of the topological flat bands as shown in Supplementary Figure 12. Each set of flat bands (d_{xz}/d_{yz} or d_{xy}/d_{x2-y2}) are doubly degenerate, and initial seeding functions are two wave functions localized at the hexagon centers with the weights distributed on the hexagon corner sites. The wave function at each site is a direct product of the spin and orbital component. To simplify the notation, we use a two component vector to represent the orbital wave function, $[\psi_{dxz}, \psi_{dyz}]$ and $[\psi_{dxy}, \psi_{dx2-y2}]$ respectively, and $[\chi_\uparrow, \chi_\downarrow]$ for the spinor wave function at each site. The hexagon corner sites are indexed as in Supplementary Figure 12a. With these notations, the Wannier seeding functions $w_\alpha = \frac{1}{\sqrt{6}}\sum_{n=1}^{6} |\psi^{(n)}\rangle \otimes \left|\chi_\alpha^{(n)}\right\rangle$ with Wannier function index α = A, B can be written as:

$$|\psi^{(n)}\rangle = \begin{bmatrix} \frac{1}{2} & \frac{-\sqrt{3}}{2} \\ \frac{\sqrt{3}}{2} & \frac{1}{2} \end{bmatrix}^{n-1} \begin{bmatrix} 0 \\ 1 \end{bmatrix} \tag{2}$$

and spinor wave function

$$\left|\chi_A^{(n)}\right\rangle = \frac{1}{\sqrt{2}}\begin{bmatrix} 1 \\ ie^{i(n-1)\pi/3} \end{bmatrix}, \quad \left|\chi_B^{(n)}\right\rangle = \frac{1}{\sqrt{2}}\begin{bmatrix} -ie^{-i(n-1)\pi/3} \\ 1 \end{bmatrix}. \tag{3}$$

The Wannier projection procedure is briefly described as follows. At momentum $\vec{k}$, the Hilbert space for the flat bands defines a projector $P^{(f)}(\vec{k}) = \sum_{i=1}^{2} \left|\psi_i^{(f)}(\vec{k})\right\rangle \left\langle\psi_i^{(f)}(\vec{k})\right|$ for either

the d_{xz}/d_{yz} or the d_{xy}/d_{x2-y2} flat band manifold. The above initial Wannier seeding functions $|\omega_\alpha(\vec{R})\rangle$ in the real space centered at the hexagon centers $\vec{R}$ can be Fourier transformed into reference states in the momentum space

$$|\omega_\alpha(\vec{k})\rangle = N\sum_{\vec{R}} e^{i\vec{k}\cdot\vec{R}}|\omega_\alpha(\vec{R})\rangle \tag{4}$$

with a proper normalization factor N. Such reference states are then used to span the flat band Hilbert space from $P^{(f)}(\vec{k})|\omega_\alpha(\vec{k})\rangle$. With the singular value decomposition (SVD), a proper set of orthonormal basis $|\omega_\alpha{}'(\vec{k})\rangle$ can be derived from the above projection of Wannier seeding functions. For the CoSn flat bands manifold, we have examined the above projection to be non-singular and the associated real-space Wannier functions can be derived from the new orthonormal basis by performing the Fourier transformation.

$$|\omega_\alpha{}'(\vec{R})\rangle = N'\int d^2\vec{k}e^{-i\vec{k}\cdot\vec{R}}|\omega_\alpha{}'(\vec{k})\rangle. \tag{5}$$

In the following section, we will examine the physical properties of these derived Wannier functions.

E. CoSn flat bands with spin-orbit coupling: Wannier function properties

We first investigate the symmetry properties of the constructed Wannier functions in detail. As discussed above, due to the topological/symmetry obstructions in constructing Wannier functions for the flat bands, we choose to abandon the inversion P, time-reversal T and mirror M_{xy} symmetries. Similarly, one has to give up two-fold rotations C_{2x} and C_{2y} as well since they can be combined with M_{xz} or M_{yz} symmetry to give rise to forbidden mirror M_{xy} symmetry operation. Instead, the constructed Wannier function satisfies C_{6z} rotational symmetry, M_{xz} or M_{yz} symmetry, combined PT symmetry, and combined $M_{xy}T$. Each flat band has two-fold degeneracy due to combined inversion and time-reversal symmetry of the system. We denote the two Wannier states in the Wannier manifolds for each flat band as ω_A and ω_B. In Supplementary Table 2, 3 we tabulate the symmetry transformation properties of these two Wannier states of each flat band group.

O	C_{6z}	M_{xz}	M_{yz}	PT	$M_{xy}T$
ω_A	$e^{-i\pi/6}\omega_A$	$-\omega_B$	$-i\omega_B$	$-\omega_B$	$i\omega_B$
ω_B	$e^{i\pi/6}\omega_B$	ω_A	$-i\omega_A$	ω_A	$i\omega_A$

Supplementary Table 2. The symmetry properties of the Wannier orbitals derived from d_{xz}/d_{yz} flat bands.

O	C_{6z}	M_{xz}	M_{yz}	PT	$M_{xy}T$
ω_A	$-e^{-i\pi/6}\omega_A$	ω_B	$-i\omega_B$	$-\omega_B$	$-i\omega_B$
ω_B	$-e^{i\pi/6}\omega_B$	$-\omega_A$	$-i\omega_A$	ω_A	$-i\omega_A$

Supplementary Table 3. The symmetry properties of the Wannier orbitals derived from d_{xy}/d_{x2-y2} flat bands.

After constructing the Wannier basis functions, one can construct the effective tight-binding Hamiltonian for the flat bands. First we would like to argue that in the two band model for a set of flat bands degenerate from the inversion symmetry, the coupling between different types of Wannier orbitals (ω_A and ω_B) always vanishes. One way to see this is that, at a given $\vec{k}$ point, the two bands are exactly degenerate. The effective two band Hamiltonian can only be a 2×2 matrix proportional to an identity matrix. Hence, the off-diagonal elements are zero at any $\vec{k}$. After Fourier transformation to the real space, the coupling between different orbital types can only be zero. The other way to see this is based on the PT symmetry properties of the Wannier states as following:

$$\begin{aligned} &\sum_{\vec{R}} tc_A^\dagger(\vec{R})c_B(0) + t^* c_B^\dagger(0)c_A(\vec{R}) \\ &\rightarrow \sum_{\vec{R}} -t^* c_B^\dagger(-\vec{R})c_A(0) - tc_A^\dagger(0)c_B(-\vec{R}) \\ &\rightarrow -\sum_{\vec{R}} tc_A^\dagger(\vec{R})c_B(0) + t^* c_B^\dagger(0)c_A(\vec{R}) \end{aligned} \tag{6}$$

Hence, such off-diagonal term has to vanish.

Due to the zero off-diagonal elements for the coupling, the model can be viewed as an effective one-band model with an emergent triangular lattice. Then the tight-binding model of the

flat band is given by the hoppings between Wannier sites as shown in Supplementary Figure 13a. The hopping terms $t_{\vec{R}}^{\omega_i}$ of the one-band model can be derived directly from the Fourier transformation of the flat band dispersion $\epsilon(\vec{k}) = \sum_{\vec{R}} t_{\vec{R}}^{\omega_i} e^{-i\vec{k}\cdot\vec{R}}$. The derived hopping parameters up to the third nearest neighbor are shown in the Supplementary Table 4. In Supplementary Figure 13b we show the effective tight-binding band dispersion for both set of flat bands overlapped with the DFT band structure.

	ϵ_0	t_1	t_2	t_3
d_{xz}/d_{yz} TBH	–435	14.3	–10.8	7.0
d_{xy}/d_{x2-y2} TBH	–273	–11.9	8.9	–14.9

Supplementary Table 4. The effective tight-binding model for the flat bands. ϵ_0 is the effective on-site energy, and t_i are couplings to the ith nearest neighbor sites. The units are in meV.

So far, we have constructed the Wannier orbitals for the group d_{xz}/d_{yz} and d_{xy}/d_{x2-y2} flat bands separately. To gain physical insights of these Wannier objects, we first investigate the actual orbital content of the derived localized wave function. The projected electron densities in Co *d* orbitals and Sn *s/p* orbitals are shown in Supplementary Table 5. The dominant component accounts for about 85% of the charge density. The mixing of d_{xy}/d_{x2-y2} in d_{xz}/d_{yz} Wannier states (and the mixing of d_{xz}/d_{yz} in d_{xy}/d_{x2-y2} Wannier states) can be expected from the band hybridization due to spin-orbit coupling as seen in the band orbital analysis near M point, which introduces additional orbital mixing.

On the other hand, the spin texture can be extracted by computing the expectation value of $\vec{S} \otimes \mathbb{I}_{orb}$ with $\mathbb{I}_{orb}$ the identity operator for the orbital degrees of freedom of each site. The spin textures of the Wannier functions are shown in Supplementary Figure 6. The spin texture of only one of the two Wannier function manifolds (say ω_A) are displayed. In case of ω_B the out-of-plane spin direction is flipped while the in-plane spin texture remains same with ω_A.

	Co d_{z2}	Co $d_{xz/yz}$	Co $d_{xy/x2\text{-}y2}$	Sn *s/p*
d_{xz}/d_{yz} Wannier orbitals	0.1 %	86.0 %	12.9 %	1.0 %
d_{xy}/d_{x2-y2} Wannier orbitals	0.9 %	7.8 %	85.4 %	5.9 %

Supplementary Table 5. The orbital content for the Wannier orbitals derived from the set of d_{xz}/d_{yz} and $d_{xy}/d_{x2\text{-}y2}$ flat bands. The orbital character is represented by the projected charge density in Co *d* orbitals and Sn *s/p* orbitals.

Next, we investigate the real space orbital textures of the flat band Wannier states. Based on the above analysis on the orbital contents and spin textures, we will focus only on the dominant orbital component for each flat band. Due to the orbital and spin entanglement on each site, visualizing the orbital content is more subtle. Below, we will present the analysis procedure for the d_{xz}/d_{yz} flat band as an example. The analysis for the d_{xy}/d_{x2-y2} Wannier states can be carried out similarly and we will just present the results. If we only consider the d_{xz}/d_{yz} orbitals and the associated spin degrees of freedom on each Co site (accounting ~ 85% of the total charge density as discussed above), the Wannier function can be represented by four component wavefunction on each site. With the Schmidt decomposition (reduced density matrix by tracing out either the spin or orbital degrees of freedom), such entangled wave function can be decomposed into the sum of two product wave functions of the orbital and spin part.

$$|\psi(\vec{r})\rangle = \left|\phi_1^{(orb)}(\vec{r})\right\rangle \otimes |\chi_1(\vec{r})\rangle + \left|\phi_2^{(orb)}(\vec{r})\right\rangle \otimes |\chi_2(\vec{r})\rangle \tag{7}$$

with a larger norm $\left|\left|\phi_1^{(orb)}(\vec{r})\right\rangle \otimes |\chi_1(\vec{r})\rangle\right|$ for the first component. $\left|\phi_i^{(orb)}(\vec{r})\right\rangle$ ($|\chi_i(\vec{r})\rangle$) are orthogonal with each other. We call the first (second) product wave function as the majority (minority) spin channel. The two spins $|\chi_i\rangle$ are anti-parallel with each other at each site. With two angles θ and ϕ defining the spin orientations, we have used the gauge convention

$$|\chi_N(\theta,\phi)\rangle = \begin{bmatrix} \cos(\theta/2) \\ \sin(\theta/2)e^{i\phi} \end{bmatrix}, \qquad |\chi_S(\theta,\phi)\rangle = \begin{bmatrix} \cos(\theta/2)e^{-i\phi} \\ \sin(\theta/2) \end{bmatrix}, \tag{8}$$

for the Wannier orbital with $\langle s_z\rangle > 0$ and $\langle s_z\rangle < 0$ respectively to avoid the singularity. The orbital component $\left|\phi_1^{(orb)}(\vec{r})\right\rangle$ ($\left|\phi_2^{(orb)}(\vec{r})\right\rangle$) corresponding to the majority (minority) spin

component on each site can be further decomposed into $\left|\phi_i^{(orb)}(\vec{r})\right\rangle = \alpha_i|d_{xz}\rangle + \beta_i|d_{yz}\rangle$ with complex coefficients α_i and β_i. To visualize the orbital content of the Wannier orbital, we also considered the real and imaginary parts separately. In our gauge convention, the dominant component is the real part orbital wave function in the majority spin channel as demonstrated in Supplementary Table 6. The orbital texture of this channel is displayed Fig. 3e,f. The resemblance with the orbital textures derived in Section A-C (Supplementary Figure 10,11) can be readily noticed.

	d_{xz}/d_{yz} Wannier states (86.0 %)	d_{xy}/d_{x2-y2} Wannier states (85.4 %)
$\left\|Re\left[\left\|\phi_1^{(orb)}(\vec{r})\right\rangle \otimes \|\chi_1(\vec{r})\rangle\right]\right\|$	85.5 %	81.4 %
$\left\|Im\left[\left\|\phi_1^{(orb)}(\vec{r})\right\rangle \otimes \|\chi_1(\vec{r})\rangle\right]\right\|$	4.4 %	10.4 %
$\left\|\left\|\phi_2^{(orb)}(\vec{r})\right\rangle \otimes \|\chi_2(\vec{r})\rangle\right\|$	10.1 %	8.2 %

Supplementary Table 6. The spin-orbital decomposition at each site into majority and minority spin channels. Within the majority spin component, the orbital component is further decomposed into real and imaginary part. The percentage shows the relative weights in each sector. This is the further refinement of decomposition for the dominant content in Supplementary Table 5.

Supplementary Note 4. Topological properties: Z_2 index

In the *s*-wave kagome lattice, the spin-orbit coupling is known to induce topological insulator phase of the isolated flat bands. Here we would like to examine the topological characters of the *d*-orbital flat bands in the presence of the spin-orbit coupling. For simplicity, we focus on the quasi-2D band structure at $k_z = 0$. The two sets of isolated flat bands can be identified over the two-dimensional Brillouin zone, separated from all the other bands. In this time-reversal invariant system with inversion symmetry, the parity eigenvalues at the time-reversal invariant momentum can be used to infer the Z_2 topological index on this plane, known as the Fu-Kane parity formula.[4] We examined the flat band states at Γ and M time-reversal invariant momenta under inversion. The parity eigenvalues at Γ and M equal to 1 and −1 respectively for both set of flat bands (see Supplementary Table 1), indicating their topologically non-trivial nature.

Apart from the bulk CoSn crystal, we are also interested in the isolated two-dimensional kagome layer crystal. The electronic structure as shown in Supplementary Figure 9 also features several sets of flat bands near the Fermi level. The system is still inversion symmetric and the flat bands are identified to be non-trivial from their parity eigenvalues.

Supplementary References

1. Bergman, D. L., Wu, C. & Balents, L. Band touching from real-space topology in frustrated hopping models, *Physical Review B* **78**, 125104 (2008).
2. Marzari, N., Mostofi, A. A., Yates, J. R., Souza, I. & Vanderbilt, D. Maximally localized wannier functions: Theory and applications, *Review of Modern Physics*, **84**, 1419-1475 (2012).
3. Soluyanov, A. A. & Vanderbilt, D. Wannier representation of Z2 topological insulators, *Physical Review B* **83**, 035108 (2011).
4. Fu, L. & Kane, C. L. Topological insulators with inversion symmetry, *Physical Review B* **76**, 045302 (2007).
5. Alexandradinata, A., Dai, X. & Bernevig, B. A. Wilson-loop characterization of inversion-symmetric topological insulators, *Physical Review B* **89**, 155114 (2014).
6. Ye, L. *et al.* Massive Dirac fermions in a ferromagnetic kagome metal, *Nature* **555**, 638–642 (2018).
7. Kang, M. *et al.* Dirac fermions and flat bands in ideal kagome metal FeSn, *Nature Materials* **19**, 163-169 (2020).
8. G. Y. Guo, S. Murakami, T.-W. Chen & N. Nagaosa. Intrinsic spin hall effect in platinum: First-principles calculations, *Phys. Rev. Lett.*, **100,** 096401 (2008).
9. G. Y. Guo, Yugui Yao & Qian Niu. *Ab initio* calculation of the intrinsic spin hall effect in semiconductors. *Phys. Rev. Lett.,* **94,** 226601 (2005).
10. Jiao, L. *et al.* Signatures for half-metallicity and nontrivial surface states in the kagome lattice Weyl semimetal $Co_3Sn_2S_2$. Phys. Rev. B **99**, 245158 (2019).
11. Yin, J.-X. *et al.* Negative flat band magnetism in a spinorbit- coupled correlated kagome magnet, *Nature Physics* **15**, 443–448 (2019).
12. Lin, Z. *et al.* Flatbands and Emergent Ferromagnetic Ordering in Fe3Sn2 Kagome Lattices, *Physical Review Letters* **121**, 096401 (2018).
