# Supplementary material for: Topological flat bands in frustrated kagome lattice CoSn
Source: Nat Commun. 2020 Aug 10;11:4004. doi: 10.1038/s41467-020-17465-1 (PMC7417556; doi:10.1038/s41467-020-17465-1)
Supplement: Supplementary file 2 — Peer Review File [file 41467_2020_17465_MOESM2_ESM.pdf]

Editorial Note: This manuscript has been previously reviewed at another journal that is not operating a transparent peer review scheme. The manuscript was considered suitable for publication without further review at Nature Communications .
